# Effects of quorum sensing–interfering agents, including macrolides and furanone C-30, and an efflux pump inhibitor on nitrosative stress sensitivity in Pseudomonas aeruginosa

**DOI:** 10.1099/mic.0.001464

**Published:** 2024-06-20

**Authors:** Shin Suzuki, Yuji Morita, Shota Ishige, Kiyohiro Kai, Kenji Kawasaki, Kazuyuki Matsushita, Kohei Ogura, Tohru Miyoshi-Akiyama†, Takeshi Shimizu

**Affiliations:** 1 Department of Molecular Infectiology, Graduate School of Medicine, Chiba University, 1-8-1 Inohana, Chiba, 260-8670, Japan; 2 Division of Laboratory Medicine, Chiba University Hospital, 1-8-1 Inohana, Chiba, 260-8677, Japan; 3 Department of Infection Control Science, Meiji Pharmaceutical University, 2-522-1 Noshio, Kiyose, Tokyo, 204-8588, Japan; 4 Laboratory of Basic and Applied Molecular Biotechnology, Division of Food Science and Biotechnology, Graduate School of Agriculture, Kyoto University, Gokasho, Uji, Kyoto, 6110011, Japan; 5 Pathogenic Microbe Laboratory, Research Institute, National Center for Global Health and Medicine, 1-21-1 Toyama, Shinjuku-ku, Tokyo, 162-8655, Japan

**Keywords:** efflux pump, efflux pump inhibitor, *P. aeruginosa*, quorum sensing–interfering agent, macrolide, nitrosative stress

## Abstract

Long-term administration of certain macrolides is efficacious in patients with persistent pulmonary *Pseudomonas aeruginosa* infection, despite how limited the clinically achievable concentrations are, being far below their MICs. An increase in the sub-MIC of macrolide exposure-dependent sensitivity to nitrosative stress is a typical characteristic of *P. aeruginosa*. However, a few *P. aeruginosa* clinical isolates do not respond to sub-MIC of macrolide treatment. Therefore, we examined the effects of sub-MIC of erythromycin (EM) on the sensitivity to nitrosative stress together with an efflux pump inhibitor (EPI) phenylalanine arginyl β-naphthylamide (PAβN). The sensitivity to nitrosative stress increased, suggesting that the efflux pump was involved in inhibiting the sub-MIC of macrolide effect. Analysis using efflux pump-mutant *P. aeruginosa* revealed that MexAB-OprM, MexXY-OprM, and MexCD-OprJ are factors in reducing the sub-MIC of macrolide effect. Since macrolides interfere with quorum sensing (QS), we demonstrated that the QS–interfering agent furanone C-30 (C-30) producing greater sensitivity to nitric oxide (NO) stress than EM. The effect of C-30 was decreased by overproduction of MexAB-OprM. To investigate whether the increase in the QS–interfering agent exposure-dependent sensitivity to nitrosative stress is characteristic of *P. aeruginosa* clinical isolates, we examined the viability of *P. aeruginosa* treated with NO. Although treatment with EM could reduce cell viability, a high variability in EM effects was observed. Conversely, C-30 was highly effective at reducing cell viability. Treatment with both C-30 and PAβN was sufficiently effective against the remaining isolates. Therefore, the combination of a QS–interfering agent and an EPI could be effective in treating *P. aeruginosa* infections.

## Introduction

*Pseudomonas aeruginosa* is a highly important bacterial pathogen in patients with chronic pulmonary diseases such as diffuse panbronchiolitis (DPB) and cystic fibrosis (CF) [1]. DPB is a pulmonary disease characterized by chronic inflammation of the bronchioles and the infiltration of inflammatory cells into the lungs, and has several features in common with CF [2]. Though the MICs of macrolides for most *P. aeruginosa* strains range between 128–512 µg ml^−1^, several studies have highlighted the benefit of long-term and low-dose macrolide treatment in patients with DPB or CF [3,4] and suggests a potential role for the maintenance of such macrolide therapy to treat these chronic pulmonary conditions. The beneficial effects of long-term, low-dose macrolides are unrelated to their antimicrobial properties. The mechanisms through which sub-MIC of macrolides affect the outcome of chronic infections with *P. aeruginosa* could include anti-inflammatory activity and modulation of bacterial virulence-factor production [5,7]. In the former, although the underlying mechanisms have yet to be fully elucidated, growing evidence supports that macrolides, especially 14- and 15-membered aglycone ring compounds, would have anti-inflammatory and immunomodulatory properties [7]. In the later, several studies have shown that macrolides interfere with *P. aeruginosa* quorum sensing (QS) [8,13].

QS is a complex population-density-based bacterial communication system. QS controls the expression of multiple virulence factors in different bacteria, and therefore blocking QS can attenuate the virulence of pathogenic bacteria. *P. aeruginosa* has three main QS systems. The first two QS systems, LasR-LasI and RhlR-RhlI, use acyl homoserine lactones (AHLs) as signalling molecules [14]. LasI and RhlI produce N-(3-oxododecanoyl)-l-homoserine lactone and N-butanoyl homoserine lactones, respectively [15,16], which are detected by their respective receptors LasR and RhlR [17]. The third signalling system uses another type of signalling molecule, 2-heptyl-3-hydroxy-4-quinolone, which has been termed the *Pseudomonas* quinolone signal (PQS), and can affect the expression of Las- and Rhl-controlled genes [18]. LasR controls the Rhl and PQS systems within the *P. aeruginosa* hierarchy, and is an attractive target for QS inhibition [19]. Although no clinical applications currently exist, many natural compounds can be used as QS inhibitors to overcome bacterial infections and antibiotic resistance in various pathogens by interrupting or attenuating QS [18,22]. For example, the brominated-furanone derivative furanone C-30 (C-30) is a structural analogue of AHL that displays enhanced antagonistic activity against *P. aeruginosa* QS systems [23]. Furanone derivatives, including C-30, are predicted to bind to LasR at the 3OC12-HSL binding site to inactivate LasR [24,25]. However, the effects of macrolides on QS may be due to a mechanism of action other than direct interaction with the LasR protein [13].

The major mechanisms used by *P. aeruginosa* to counter antibiotic attack can be classified as intrinsic, acquired, or adaptive. *P. aeruginosa* possesses a high level of intrinsic resistance to most antibiotics through restricted outer-membrane permeability, efflux systems that pump antibiotics from the cell, and antibiotic-inactivating enzymes. *P. aeruginosa* is sensitive to a limited number of drugs, including several β-lactams and aminoglycosides. However, several strains of *P. aeruginosa* that are resistant to these antibiotics have emerged, such as multidrug-resistant *P. aeruginosa* (MDRP), which has become a significant public health concern, as few, and sometimes even no, effective antimicrobial agents are available for treating *P. aeruginosa* infection.

Efflux-mediated antibiotic resistance in *P. aeruginosa* is primarily conferred by efflux pumps belonging to the resistance/nodulation/division (RND) superfamily [26]. The major RND efflux pumps of clinical importance in *P. aeruginosa* are MexAB-OprM, MexCD-OprJ, MexEF-OprN, and MexXY-OprM, which differ in expression patterns and substrate specificities [27,29]. MexAB-OprM is a constitutively expressed primary efflux pump in *P. aeruginosa* and is responsible for most of the efflux activity [30,31]. MexAB-OprM is multiply regulated in *P. aeruginosa*. Three transcriptional regulators have been identified: MexR, NalC, and NalD [32,35]. MexR is a major regulator directly repressing MexAB-OprM expression [32]. The MexR-DNA interaction is also inhibited by ArmR, the expression of which is regulated by NalC [35,36]. NalD is also a negative transcriptional regulator of the *mexAB-oprM* operon [34,37]. The deletion of this pump renders *P. aeruginosa* highly susceptible to β-lactam antibiotics, whereas its overexpression increases antibiotic resistance [30,38]. MexCD-OprJ, MexEF-OprN, and MexXY-OprM expel β-lactams [39], quinolones [40], and aminoglycosides [41,43], respectively. Overproduction of multiple efflux pumps has been observed in several clinical strains of *P. aeruginosa*, broadening bacterial resistance to antibiotics and contributing to development of multidrug resistance [44,46]. Using an efflux pump inhibitor (EPI) improves the treatment efficacy of antibiotic therapy for treating *P. aeruginosa* infections [26,47,51]. Phenylalanine arginyl β-naphthylamide (PAβN) is a well-studied EPI that impairs antibiotic efflux through competitive inhibition of efflux pumps. PAβN is broadly inhibitive against MexAB-OprM, MexCD-OprJ, MexEF-OprN and MexXY-OprM in *P. aeruginosa* [52] and can be used as a diagnostic tool to detect active efflux as a mechanism of resistance in pathogens.

NO is an essential component of the innate immune system against infection, and it is produced by host defence mechanisms such as neutrophils and activated macrophages [53,54]. NO is also produced as a by-product of bacteria ammonia production by nitrite reduction [55,56]. The physiological concentrations of NO within the gut are believed to between 10^−7^ M–10^−6^ M [57,58]. Therefore, bacteria can potentially be exposed to various concentrations of NO produced by host cells and endogenously generated NO. Exhaled NO from patients with CF is lower than that produced by normal patients [59,61]. Reduced NO production in CF may account for hypersusceptibility to infection by opportunistic bacteria.

We previously reported that an increase in the macrolide exposure-dependent sensitivity of *P. aeruginosa* to nitrosative stress is a typical characteristic of *P. aeruginosa,* including MDRP [62]. However, a few *P. aeruginosa* strains did not respond to macrolide treatment, suggesting that the efflux pump may be associated with a reduction in the macrolide effect.

In this study, we showed that the QS–interfering agent C-30 had a greater effect on the sensitivity of *P. aeruginosa* against nitrosative stress than erythromycin (EM). The effect of EM was mainly decreased by the production of MexAB-OprM and MexXY-OprM, whereas that of C-30 was decreased by the overproduction of MexAB-OprM. Moreover, we found that the effects of EM and C-30 on nitrosative stress were enhanced by PAβN.

## Methods

### Bacterial strains, plasmids, media and chemicals

Wild-type *P. aeruginosa*, gene-disrupted derivatives, and plasmids carrying Mex genes used in this study are shown in Table 1*. Escherichia coli* DH5α and ME9806 cells were used as hosts for plasmid construction. *E. coli* S17-1 was used as a mobilizer strain. Bacterial cells were grown in Luria–Bertani (LB) broth at 37 °C unless otherwise indicated. pEX18Tc and their derivatives were maintained and selected in *E. coli* using medium supplemented with 10 µg ml^−1^ tetracycline at 25–30 °C for 36–48 h. pFLP2 and pPS858 were maintained and selected using medium supplemented with 100 µg ml^−1^ ampicillin for *E. coli* or 200 µg ml^−1^ carbenicillin for *P. aeruginosa*. pUCP20T and their derivatives were maintained and selected using medium supplemented with 50 µg ml^−1^ ampicillin for *E. coli* or 50 µg ml^−1^ carbenicillin for *P. aeruginosa* YM64. EM was purchased from Sigma-Aldrich (St. Louis, MO, USA). NO was generated using the NO donor DETA NONOate (DETA/NO) (Cayman Chemical Company, MI, USA). The half-life of DETA/NO is 20 h at 37 °C in 0.1 M phosphate buffer (pH 7.4). PAβN and C-30 were obtained from Cayman Chemical Company and AdipoGen LIFE SCIENCES (CA, USA), respectively.

**Table 1.** Bacterial strains and plasmids

Strains	Relevant characteristics	References
*P. aeruginosa* strains		
PAO1	*P. aeruginosa*, wild type	[96]
PRoprD1	PAO1 derivative with *oprD: :FRT*	This study
YM24	PAO1 derivative with *mexCD-oprJ: :FRT, mexEF-oprN: :FRT, mexXY: :FRT*	[68]
YM34	PAO1 derivative with *mexAB: :FRT, mexCD-oprJ: :FRT, mexEF-oprN: :FRT*	[68]
YM62	PAO1 derivative with *mexAB-oprM: :FRT, mexCD-oprJ: :FRT, mexXY: :FRT*	[68]
YM63	PAO1 derivative with *mexAB-oprM: :FRT, mexEF-oprN: :FRT, mexXY: :FRT*	[68]
YM64	PAO1 derivative with *mexAB-oprM: :FRT, mexCD-oprJ: :FRT, mexEF-oprN: :FRT, mexXY: :FRT*	[68]
86nfxBG1	YM63 derivative with *nfxB::aacC-gfp* cassette flanked by *FRT*	This study
PRmexR2	PAO1 derivative with *mexR: :FRT*	This study
PRnalD1	PAO1 derivative with *nalD: :FRT*	This study
GUPR731	multidrug-resistant *P. aeruginosa*, ST 3390	[62]
GUPR800	multidrug-resistant *P. aeruginosa*, ST 235	[62]
GUPR801	multidrug-resistant *P. aeruginosa*, ST 235	[62]
GUPR810	multidrug-resistant *P. aeruginosa*, ST 357	[62]
GUPR824	multidrug-resistant *P. aeruginosa*, ST 1930	[62]
GUPR848	multidrug-resistant *P. aeruginosa*, ST 235	[62]
GUPR850	multidrug-resistant *P. aeruginosa*, ST 1197	[62]
GUPR852	multidrug-resistant *P. aeruginosa*, ST 235	[62]
GUPS884	non-multidrug-resistant *P. aeruginosa*, ST 1817	[62]
GUPS885	non-multidrug-resistant *P. aeruginosa*, ST 244	[62]
GUPS899	non-multidrug-resistant *P. aeruginosa*, ST 1816	[62]
*E. coli* strains		
DH5α	Φ80d *lacZ∆M15 endA1 recA1 hsdR17(r*_*K*_^*-*^ *m*_*K*_^*+*^*) supE44 thi-1 gyrA96 relA1 F*^*-*^*∆ (lacZYA-argF)U169*	National BioResource Project (NIG,Japan)
ME9806	∆*hsdR* ∆*endA* ∆*recA*	[63]
S17-1	*recA thi pro hsdR*- Tra^+^	[97]
Plasmids	Relevant characteristics	
pUCP20T	mobilizable broad-host-range expression vector, *lac* promoter, Amp^r^	[98]
pmexAB1	pUCP20T derivative carrying *mexAB-oprM* gene	This study
pmexCD1	pUCP20T derivative carrying *mexCD-oprJ* gene	This study
pmexEF3	pUCP20T derivative carrying *mexEF-oprN* gene	This study
pmexXY1	pUCP20T derivative carrying *mexXY-oprM* gene	This study
pEX18Tc	Broad-host-range gene replacement vector; *sacB* Tet^r^	[99]
pPS858	Source of *aacC1*, *gfp* cassette flanked by *FRT*	[99]
pEXoprG3	pEX18Tc derivative carrying *oprD::aacC1, gfp* cassette flanked by *FRT*	This study
pEXnfxG2	pEX18Tc derivative carrying *nfxB::aacC1, gfp* cassette flanked by *FRT*	This study
pEXnalDG1	pEX18Tc derivative carrying *nalD::aacC1, gfp* cassette flanked by *FRT*	This study
pExmexRG2	pEX18Tc derivative carrying *mexR::aacC1*, gfp cassette flanked by *FRT*	This study
pFLP2	Source of Flp recombinase; *sacB* Amp^r^	[99]

Amprampicillin-resistantTetrtetracycline-resistant

### NO sensitivity assays

*P. aeruginosa* was grown in LB broth, with EM, C-30, and/or PAβN as needed, at 37 °C for 18 h. Since the MIC of EM for *P. aeruginosa* PAO1 was 256 µg ml^−1^, we used 10 µg ml^−1^ of EM as sub-MIC. Bacteria were rinsed three times with saline solution containing 0.1 % glucose, 10 mM MOPS (pH 7.0), and the same concentration of EM, C-30, and/or PAβN. Bacterial suspensions were diluted 1 : 100 with saline solution containing 0.1 % glucose, 10 mM MOPS (pH 7.0), and the same concentration of EM, C-30, and/or PAβN with or without 100 µM DETA/NO, and were then incubated at 37 °C for 22 h. The number of viable bacteria was determined using bacterial plate count.

### Plasmid constructions

#### Constructions of Mex-expression plasmids

To construct a plasmid expressing MexAB-OprM, MexCD-OprJ, and MexEF-OprN, DNA fragments of *mexAB-oprM*, *mexCD-oprJ,* and *mexEF-oprN* were PCR amplified from the genomic DNA of *P. aeruginosa* PAO1 strain using primer sets P1486–P1487, P1482–P1483, and P1484–P1485, respectively. To construct a plasmid expressing MexXY-OprM, DNA fragments of *mexXY* and *oprM* were PCR amplified from the genomic DNA of the PAO1 strain using the primer sets P1488–P1489 and P1490–P1487, respectively. A linear DNA fragment was obtained by PCR from the plasmid DNA of pUCP20T using primer set P1396–P1397. These DNA fragments were cloned into pUCP20T using the iVEC system [63] or In-fusion HD cloning kit (TAKARA, Tokyo, Japan) to yield pmexAB1, pmexCD1, pmexEF3, and pmexXY1 (Table 1). Constructs were confirmed by restriction digestion and DNA sequencing. The expression plasmid was introduced into the integrants via conjugational transfer. The conjugational integrants were selected on LB agar containing carbenicillin (50 µg ml^−1^) and chloramphenicol (2 µg ml^−1^) at 37 °C for 18 h.

#### Construction of deletion plasmids

To introduce deletions of *oprD*, *nfxB*, *mexR*, and *nalD* into *P. aeruginosa*, deletion constructs were prepared in plasmid pEX18Tc by cloning PCR-amplified DNA fragments corresponding to the regions upstream of the target genes, the gentamicin-resistance (Gm^r^)-selectable FRT cassette, and downstream regions of the target gene to be deleted. The upstream and downstream regions of *oprD*, *nfxB*, *mexR,* and *nalD* were PCR amplified from the genomic DNA of *P. aeruginosa* PAO1 using primer sets P1565–P1566, P1431–P1435, P1438–P1446, and P1636–P1637 for the upstream regions, and primer sets P1567–P1568, P1433–P1434, P1447–P1439, and P1638–P1639 for the downstream regions. The Gm^r^-selectable FRT cassette was PCR amplified from pPS858 using primer set P1394–P1395. A linear DNA fragment of pEX18Tc was obtained by PCR from pEX18Tc using primer set P1396–P1397. These DNA fragments of the regions upstream of the target genes, Gm^r^-selectable FRT cassette, and the regions downstream of the target genes were tandemly cloned into pEX18Tc using the iVEC system [63] or In-fusion HD cloning kit to yield pEXoprG3, pEXnfxG2, pExmexRG2, and pEXnalDG1 (Table 1). Constructs were confirmed by restriction digestion and DNA sequencing.

### Conjugational transfer

Plasmids were mobilized by the conjugational transfer into *P. aeruginosa* from *E. coli* S17-1. The donor *E. coli* strain harbouring plasmid was grown overnight at 37 °C with shaking while the recipient *P. aeruginosa* was grown statically overnight in LB broth containing 50 mM KNO_3_ at 42 °C. The donor and recipient were mixed at a ratio of 3 : 1, harvested by centrifugation (8,000 r.p.m., 5 min, room temperature), resuspended in 0.9 % NaCl, and spotted onto a tryptic soy agar plate. Following an incubation at 37 °C for 6 h to overnight, cells were resuspended in 2 ml of a 0.9 % NaCl solution, diluted 10-fold, and then plated onto selective LB medium containing appropriate antibiotics at 37 °C for 18 h.

### Construction of deletion mutants

The gene-deletion plasmids containing the Gm^r^-selectable FRT cassette was mobilized by conjugational transfer into *P. aeruginosa* from *E. coli* S17-1. The conjugational integrants were selected on LB agar containing tetracycline (50 µg ml^−1^), gentamicin (30 µg ml^−1^) and chloramphenicol (5 µg ml^−1^) at 37 °C for 18 h. The recovered merodiploids harbouring unwanted plasmid backbones were streaked onto a sucrose agar plate and incubated at 25–30 °C for 36–48 h. Sucrose-resistant colonies were patched on the same plate and LB agar plates containing tetracycline (50 µg ml^−1^) to confirm the loss of the plasmid backbones (tetracycline-sensitive). Colony PCR was used to confirm the appropriate deletions in the sucrose-resistant and tetracycline-sensitive clones.

Deletion of chromosomally integrated Gm^r^ markers was achieved using pFLP2. The pFLP2 vector was introduced into integrants via conjugational transfer. Conjugational integrants were selected on LB agar containing carbenicillin (200 µg ml^−1^) and chloramphenicol (5 µg ml^−1^) to cure the Gm^r^-selectable FRT cassette via the pFLP2-encoded Flp recombinase at 37 °C for 18 h. Carbenicillin-resistant colonies containing pFLP2 were subsequently streaked onto a sucrose agar plate to select for loss of pFLP2. Sucrose-resistant colonies were patched onto the same plate and LB agar plates carbenicillin (200 µg ml^−1^) and incubated at 25–30 °C for 36–48 h to confirm the loss of the pFLP2 plasmid (carbenicillin-sensitive). Colony PCR used to confirm the presence of appropriate deletions in clones.

### Bacterial mRNA analysis

*P. aeruginosa* was grown in LB broth with or without EM or C-30 at 37 °C for 8 h (log phase) or 14 h (stationary phase). Cells were pelleted by centrifugation at 10 000 ***g*** for 5 min. Total bacterial RNA was isolated using the NucleoSpin RNA Plus (MACHEREY-NAGEL GmbH and Co. KG, Duren, Germany), and the concentration was determined by measuring the A260. Subsequently, 0.5 µg of RNA from each sample was reverse-transcribed using ReverTra Ace qPCR RT Master Mix with gDNA Remover (TOYOBO, Osaka, Japan). cDNA (1 µg) was used as a template for quantitative PCR (qPCR) amplification with the designed primer sets for *mexA* (P1452–P1453) [64], *mexD* (P1464–P1465) [65], *mexE* (P1456–P1457) [64]*, mexX* (P1462–P1463) [66], and *rpsL* (P1460–P1461) [64] (Table S1, available in the online version of this article). Real-time qPCR was performed using TB Green *Premix Ex Taq* II (Tli RNase H Plus) (TAKARA, Tokyo, Japan) in a LightCycler 96 system (Roche Diagnostics, Tokyo, Japan). The protocol included initial denaturation at 95 °C for 30 s, followed by 40 cycles of amplification, comprising denaturation at 95 °C for 5 s, annealing at 60 °C (*mexA*, *mexD,* and *rpsL*) or 62 °C (*mexE* and *mexX*) for 20 s, and extension at 72 °C for 30 s. *rpsL* was used as the housekeeping gene to calculate the relative expression levels of each gene. Relative target levels were calculated using the ∆∆Ct method [67]. Results represent the average enrichment measured by qPCR in at least three replicates.

### Statistical analyses

Unpaired *t*-tests were used to determine significant differences when only two treatment groups were compared. One-way ANOVA with the Student–Newman–Keuls multiple comparisons test was used to analyse significant differences among multiple groups.

## Results

### Effect of sub-MIC of macrolide on the sensitivity to nitrosative stress of *P. aeruginosa* PAO1 treated with EPI

**Fig. 1.**
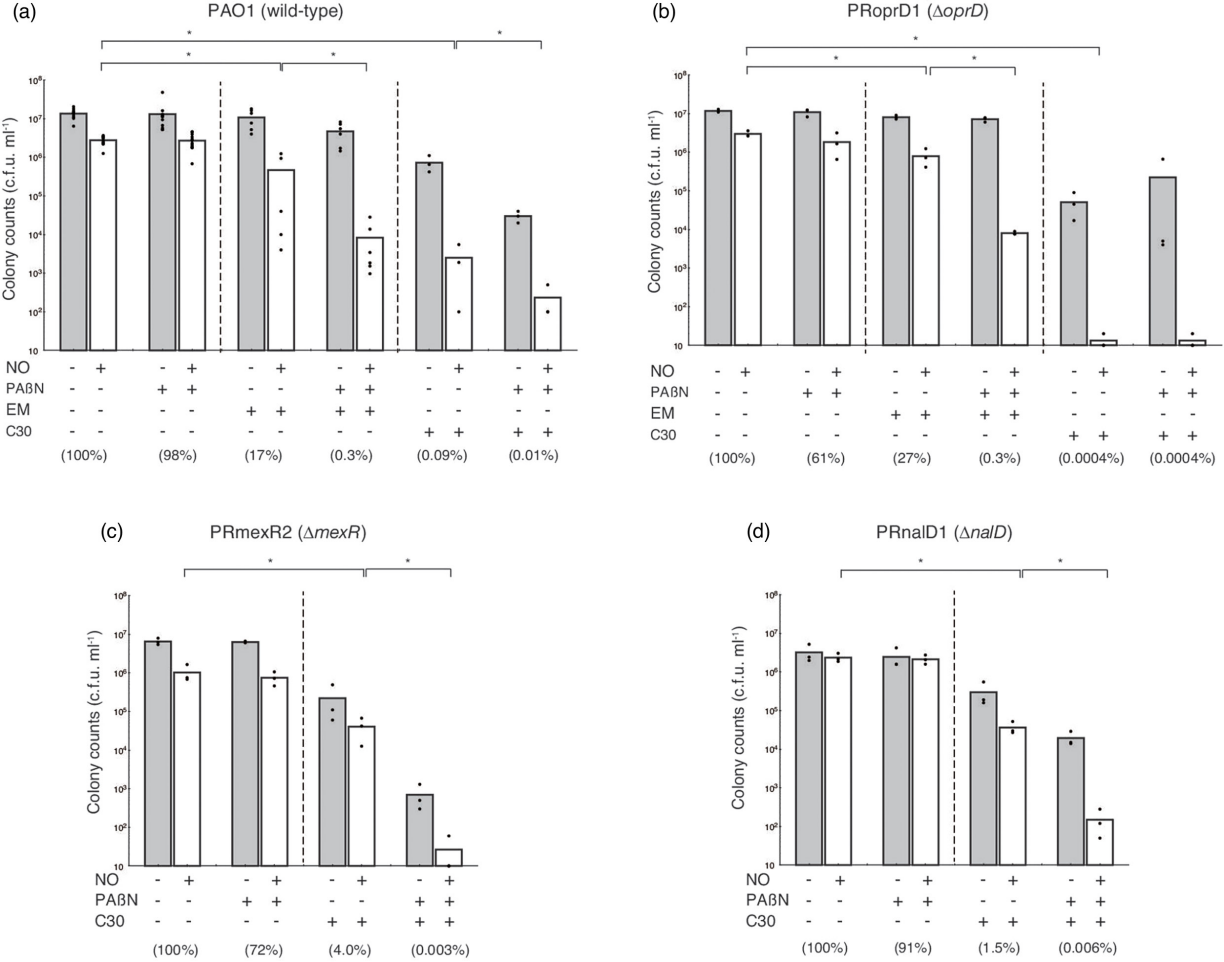
Effects of QS–interfering agents and EPI on the sensitivity to nitrosative stress in *P. aeruginosa* PAO1 and gene-disrupted derivatives. Bacteria were grown in LB broth, with 10 µg ml^−1^ EM, 10 µM C-30, and/or 10 µg ml^−1^ PAβN as needed at 37 °C for 18 h. Bacteria were rinsed with saline solution containing 0.1 % glucose, 10 mM MOPS (pH 7.0), and the same concentration of EM, C-30, and/or PAβN. Bacterial suspensions were diluted 1 : 100 with saline solution containing 0.1 % glucose, 10 mM MOPS (pH 7.0), and the same concentration of EM, C-30, and/or PAβN with or without 100 µM DETA/NO, and then were incubated at 37 °C for 22 h. The number of viable bacteria was determined using bacterial plate counts. Percentages in parentheses represent the ratio to the colony count treated with the NO donor alone. All assays were performed at least three times. Results are expressed as the means and individual data points. Unpaired *t*-tests were used to determine significant differences when only two treatment groups were compared. One-way ANOVA with Student–Newman–Keuls multiple comparisons test was used to analyse significant differences among multiple groups. Asterisks indicate a significant difference (*P<0.01). (**a**) PAO1 (wild-type); (**b**) PRoprD1 (*∆oprD*); (**c**) PRmexR2 (*∆mexR*); (**d**) PRnalD1 (*∆nalD*).

To determine the effect of low-dose macrolides on the sensitivity of *P. aeruginosa* PAO1 to nitrosative stress, the NO donor DETA/NO was used as the source of NO. *P. aeruginosa* PAO1 grown in the absence of EM in saline solution containing 0.1 % glucose, 10 mM MOPS (pH 7.0) treated with 100 µM DETA/NO produced a colony count of 2.8×10^5^ c.f.u./100 µl after 22 h (Fig. 1a). In the presence of 10 µg ml^−1^ EM, the colony count with an NO donor treatment was 4.6×10^4^ c.f.u./100 µl; the ratio of the colony count treated with the NO donor and EM was 17 % of that treated with the NO donor alone (Fig. 1a). Efflux-mediated antibiotic resistance in *P. aeruginosa* is primarily mediated by efflux pumps [26]. When *P. aeruginosa* was treated with EM, the relative expression of efflux pump genes in *P. aeruginosa* PAO1 increased (Table 2). In the presence of both EM and 10 µg ml^−1^ PAβN, the colony count treated with an NO donor was 8.3×10^2^ c.f.u./100 µl (0.3 %) (Fig. 1a). These results suggest that efflux pumps are involved in the inhibitory effects of low-dose macrolides.

**Table 2.** Relative expressions of multidrug efflux pump genes in *P. aeruginosa* under stationary phase and logarithmic

Strains	Addition	*mexAB-oprM*	*mexCD-oprJ*	*mexEF-oprN*	*mexXY-oprM*
Stationary phase					
PAO1	–	1.0	1.0	1.0	1.0
	EM	22±14	38±22	162±100	59±31
	C-30	28±13	337±217	118±37	89±21
YM24	–	1.9±0.3	–	–	–
	EM	9.9±1.7	–	–	–
	C-30	8.8±2.9	–	–	–
YM63	–	–	5.5±1.6	–	–
	EM	–	3.1±0.6	–	–
	C-30	–	33±7.2	–	–
86nfxBG1	–	–	50±12	–	–
	EM	–	49±12	–	–
YM62	–	–	–	14±4.5	–
	EM	–	–	159±44	–
	C-30	–	–	3.1±0.5	–
YM34	–	–	–	–	0.9±0.3
	EM	–	–	–	54±11
	C-30	–	–	–	28±4.9
YM64 (pmexAB1)	–	1.7±0.2	–	–	–
	EM	40±4.1	–	–	–
	C-30	58±6.6	–	–	–
YM64 (pmexCD1)	–	–	244±44	–	–
	EM	–	1027±235	–	–
	C-30	–	7380±703	–	–
YM64 (pmexEF3)	–	–	–	831±163	–
	EM	–	–	858±183	–
	C-30	–	–	4000±1300	–
YM64 (pmexXY1)	–	–	–	–	1271±268
	EM	–	–	–	127±44
	C-30	–	–	–	3962±597
Logarithmic phase					
PAO1	–	1.0	nd	nd	nd
	C-30	3.3±2.6	nd	nd	nd
PRmexR2	–	15±6.8	nd	nd	nd
	C-30	14±5.3	nd	nd	nd
PRnalD1	–	12±5.4	nd	nd	nd
	C-30	14±5.9	nd	nd	nd
PAO1	–	1.0	nd	nd	nd
	C-30	2.0±0.4	nd	nd	nd
YM64 (pmexAB1)	–	4.6±1.2	–	–	–
	C-30	17±3.4	–	–	–

ndnot determined

The outer membrane of *P. aeruginosa*, which acts as a selective barrier to prevent antibiotic penetration, is an asymmetric bilayer of phospholipid and lipopolysaccharide embedded with porins that form β-barrel protein channels. The absence of OprD increases antibiotic resistance [27]. Therefore, to confirm whether OprD is involved in the inhibition of the effects of low-dose macrolides, we used an *oprD*-deficient *P. aeruginosa* strain PRoprD1. In the presence of EM, the colony count of *P. aeruginosa* PRoprD1 treated with an NO donor was similar to that of *P. aeruginosa* PAO1 (Fig. 1b). This suggests that the outer membrane protein OprD is not involved in the inhibition of the low-dose macrolide effect.

### Effect of sub-MIC of macrolide on the sensitivity to nitrosative stress of single-efflux pump *P. aeruginosa*

To determine which efflux pumps were responsible for the reduced low-dose macrolide effect, further analysis was performed using a *P. aeruginosa* strain that only contained a single gene that expressed an efflux pump. The relative expression of the efflux pump genes was similar in wild-type PAO1 and each of the single-efflux pump *P. aeruginosa* strains (Table 2). When single-efflux pump gene-expressing strains were treated with EM, the relative expression of each efflux pump gene also increased (Table 2).

**Fig. 2.**
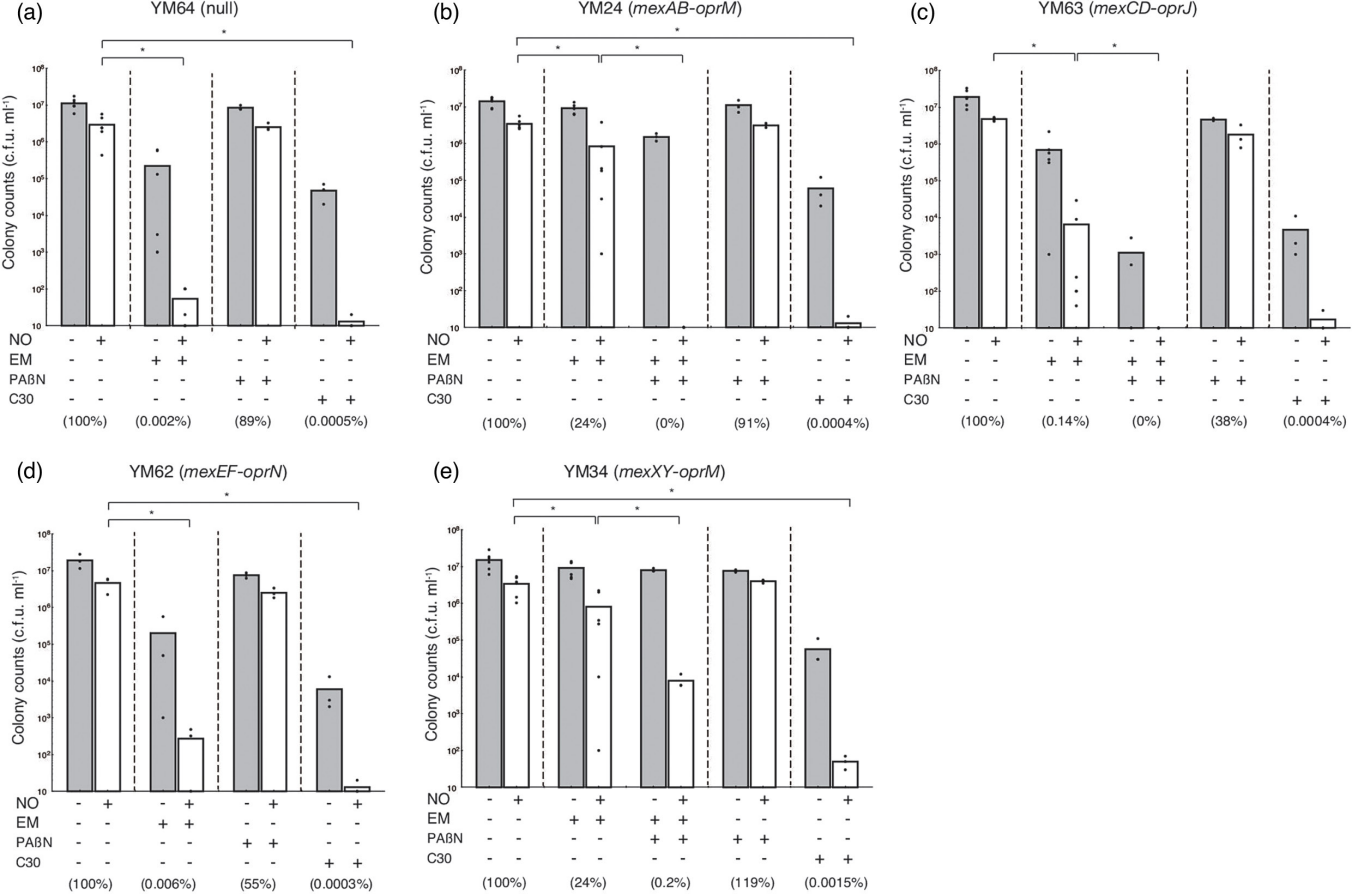
Effects of QS–interfering agents and EPI on the sensitivity to nitrosative stress of single-efflux pump *P. aeruginosa*. Bacteria were grown in LB broth, with 10 µg ml^−1^ EM, 10 µM C-30, and/or 10 µg ml^−1^ PAβN as needed at 37 °C for 18 h. Bacteria were rinsed with saline solution containing 0.1 % glucose, 10 mM MOPS (pH 7.0), and the same concentration of EM, C-30, and/or PAβN. Bacterial suspensions were diluted 1 : 100 with saline solution containing 0.1 % glucose, 10 mM MOPS (pH 7.0), and the same concentration of EM, C-30, and/or PAβN with or without 100 µM DETA/NO, and then were incubated at 37 °C for 22 h. The number of viable bacteria was determined using bacterial plate counts. Percentages in parentheses represent the ratio to the colony count treated with the NO donor alone. All assays were performed at least three times. Results are expressed as the means and individual data points. Unpaired *t*-tests were used to determine significant differences when only two treatment groups were compared. One-way ANOVA with the Student–Newman–Keuls multiple comparisons test was used to analyse significant differences among multiple groups. Asterisks indicate a significant difference (*P<0.01). (**a**) YM64 (null); (**b**) YM24 (*mexAB-oprM*); (**c**) YM63 (*mexCD-oprJ*); (**d**) YM62 (*mexEF-oprN*); (**e**) YM34 (*mexXY-oprM*).

The susceptibility of *P. aeruginosa* to many antibiotics was restored when the four systems most relevant for antibiotic resistance (MexAB-OprM, MexCD-OprJ, MexEF-OprN, and MexXY-OprM) were deleted [68]. When using null *P. aeruginosa* (YM64) that lacked the four types of efflux pump genes, in the presence of EM, the colony count treated with an NO donor was 5.4 c.f.u./100 µl (0.002 %) (Fig. 2a). In the presence of EM, the colony counts of *mexAB-oprM–*present *P. aeruginosa* (YM24) and *mexXY-oprM*–present *P. aeruginosa* (YM34) treated with an NO donor were 8.4×10^4^ and 8.1×10^4^ c.f.u./100 µl (24 and 24 %), respectively (Fig. 2b, e). Moreover, in the presence of both EM and PAβN, the colony counts for YM24 and YM34 cells treated with an NO donor decreased (Fig. 2b, e). However, PAβN effectively inhibited the efflux of EM by MexAB-OprM (Fig. 2b, e). These results indicate that low-dose macrolide effects were reduced by MexAB-OprM and MexXY-OprM to discharge EM. However, in the presence of EM, the colony counts of *mexCD-oprJ*–present *P. aeruginosa* (YM63) and *mexEF-oprN*–present *P. aeruginosa* (YM62) treated with an NO donor were 6.5×10^2^ and 2.7×10^1^ c.f.u./100 µl (0.14 and 0.006 %), respectively, indicating that MexCD-OprJ could partially pump out EM (Fig. 2c, d). These results indicate that MexAB-OprM and MexXY-OprM are factors that reduce the low-dose macrolide effect and that MexCD-OprJ retains the potential to attenuate low-dose macrolide effects.

### Effect of sub-MIC of macrolide on the sensitivity to nitrosative stress in *P. aeruginosa* overexpressing efflux pump genes

To confirm that efflux pumps were responsible for the reduced low-dose macrolide effect, four efflux pump genes were overexpressed in YM64. The expression levels of *mexCD-oprJ*, *mexEF-oprN*, and *mexXY-oprM* in the efflux pump-overexpressing *P. aeruginosa* were higher than those in the wild-type and each single-efflux pump–expressing *P. aeruginosa* strain (Table 2). However, the expression of *mexAB-oprM* in efflux pump–overexpressing *P. aeruginosa* was similar to that in the wild-type and *mexAB-oprM*–present *P. aeruginosa*, suggesting that *mexAB-oprM* in the wild-type was highly constitutively expressed under laboratory conditions.

**Fig. 3.**
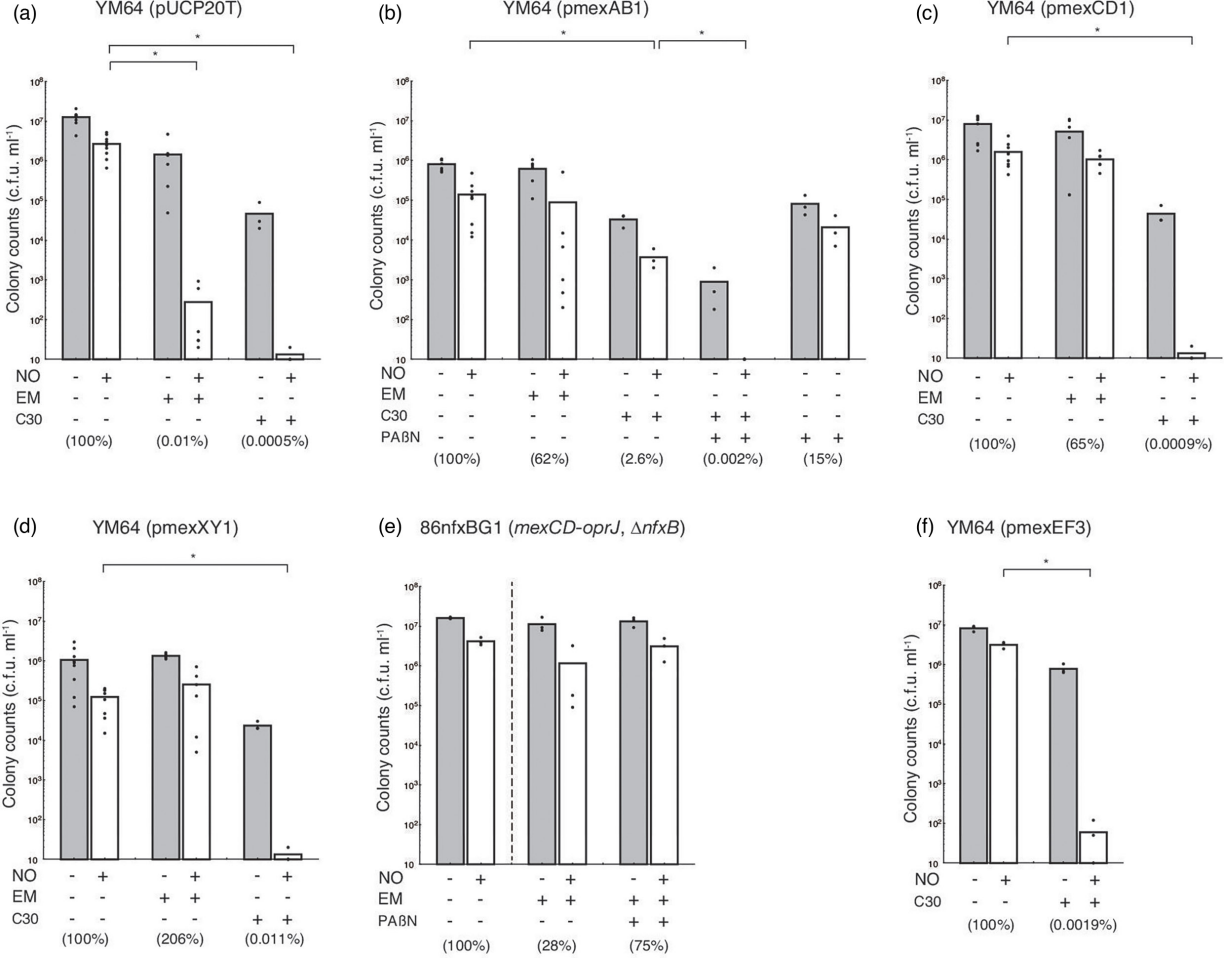
Effects of QS–interfering agents and EPI on the sensitivity to nitrosative stress in *P. aeruginosa* overexpressing efflux pump genes. Bacteria were grown in LB broth, with 10 µg ml^−1^ EM, 10 µM C-30, and/or 10 µg ml^−1^ PAβN as needed at 37 °C for 18 h. Bacteria were rinsed with saline solution containing 0.1 % glucose, 10 mM MOPS (pH 7.0), and the same concentration of EM, C-30, and/or PAβN. Bacterial suspensions were diluted 1 : 100 with saline solution containing 0.1 % glucose, 10 mM MOPS (pH 7.0), and the same concentration of EM, C-30, and/or PAβN with or without 100 µM DETA/NO, and then were incubated at 37 °C for 22 h. The number of viable bacteria was determined using bacterial plate counts. Percentages in parentheses represent the ratio to the colony count treated with the NO donor alone. All assays were performed at least three times. Results are expressed as the means and individual data points. Unpaired *t*-tests were used to determine significant differences when only two treatment groups were compared. One-way ANOVA with the Student–Newman–Keuls multiple comparisons test was used to analyse significant differences among multiple groups. Asterisks indicate a significant difference (*P<0.01). (**a**) YM64 (pUCP20T); (**b**) YM64 (pmexAB1); (**c**) YM64 (pmexCD1); (**d**) YM64 (pmexXY1); (**e**) 86nfxBG1 (*mexCD-oprJ, ∆nfxB*); (**f**) YM64 (pmexEF3).

Although YM64 containing the control vector pUCP20T could grow in LB broth with 10 µg ml^−1^ EM, *mexEF-oprN*–overexpressed *P. aeruginosa* could not grow (Fig. S1). When using YM64 containing the control vector in the presence of EM, the colony count following treatment with an NO donor was 2.8×10 c.f.u./100 µl (0.01 %) (Fig. 3a). In the presence of EM, the colony counts of *mexAB-oprM*, *mexCD-oprJ*, and *mexXY-oprM*–overexpressed *P. aeruginosa* treated with an NO donor were 8.9×10^3^, 1.0×10^5^ and 2.5×10^4^ c.f.u./100 µl (62 %, 65 %, and 206 %), respectively (Fig. 3b, c, d). Moreover, when using *nfxB*-deficient *mexCD-oprJ*–present *P. aeruginosa* (86nfxBG1) using YM63 as parent strain, in the presence of EM, the colony count following treatment with an NO donor was 1.2×10^5^ c.f.u./100 µl (28 %) (Fig. 3e). The deletion of *nfxB*, which encodes a negative regulator of *mexCD-oprJ*, in 86nfxBG1 cells increased the expression of *mexCD-oprJ* (Table 2). However, PAβN did not reduce the effect of EM on 86nfxBG1, although the effect of EM onYM63 was inhibited, suggesting that PAβN did not inhibit the efflux of EM by overproducing MexCD-OprJ (Fig. 3e). These results indicate that overproduction of MexAB-OprM, MexXY-OprM, and MexCD-OprJ reduce the low-dose macrolide effect.

### Effect of QS–interfering agent on the sensitivity to nitrosative stress of *P. aeruginosa* PAO1 and single-efflux pump *P. aeruginosa*

Macrolides exhibit extensive antagonistic activities to QS [7,11]. Therefore, to investigate whether an increase in QS–interfering agent exposure-dependent sensitivity to nitrosative stress is a typical characteristic of *P. aeruginosa*, further analysis was performed using the QS–interfering agent, C-30. In the presence of 10 µM (2.5 µg ml^−1^) C-30, the colony count of *P. aeruginosa* PAO1 treated with an NO donor was 2.5×10^2^ c.f.u./100 µl (0.09 %) (Fig. 1a, S2). To compare the effects of EM and C-30, we measured the viability of *P. aeruginosa* PAO1 in response to nitrosative stress after treatment with EM and C-30. In the presence of EM or C-30, the viability of *P. aeruginosa* PAO1 treated with the NO donor decreased in a dose-dependent manner (Fig. S2), although C-30 was more effective than EM (Fig. S2). When *P. aeruginosa* PAO1 was treated with C-30, the relative expression of efflux pump genes increased, although the expression of efflux pump genes in *P. aeruginosa* PAO1 treated with C-30 was lower than that in cells treated with EM (Table 2). In the presence of both C-30 and PAβN, the colony count of *P. aeruginosa* PAO1 treated with an NO donor was 2.3×10 c.f.u./100 µl (0.01 %) (Fig. 1a). This result suggests that efflux pump(s) diminish the effects of C-30. However, in the presence of C-30, the colony count of *P. aeruginosa* PRoprD1 treated with an NO donor was 1.3 c.f.u./100 µL (0.0004 %), indicating that the presence of OprD reduces the effect of C-30 (Fig. 1b).

To determine which efflux pumps were responsible for the reduced effect of C-30, further analysis was performed using a single-efflux pump *P. aeruginosa*. When using YM64, in the presence of C-30, the colony count following treatment with an NO donor was 1.3 c.f.u./100 µl (0.0005 %) (Fig. 2a). In the presence of C-30, the colony counts of YM24, YM63, YM62, and YM34 treated with an NO donor were 1.3–5.0 c.f.u./100 µl (0.0003 %–0.0015 %) (Fig. 2b–e). These results indicate that the effect of C-30 cannot be suppressed by the expression of a single efflux pump gene at normal levels under an endogenous promoter.

### Effect of QS–interfering agent on the sensitivity to nitrosative stress in *P. aeruginosa* overexpressing efflux pump genes

When using YM64 containing control vector, in the presence of C-30, the colony count following treatment with an NO donor was 1.3 c.f.u./100 µl (0.0005 %) (Fig. 3a). In the presence of C-30, the colony counts of *P. aeruginosa* strains overexpressing *mexAB-oprM*, *mexCD-oprJ*, *mexEF-oprN*, or *mexXY-oprM* and that were treated with an NO donor were 3.7×10^2^, 1.3, 1.3, and 6.0 c.f.u./100 µl (2.6 %, 0.0009 %, 0.011 %, and 0.0019 %), respectively (Fig. 3b, c, d, f). This result indicated that MexAB-OprM could attenuate the C-30 effect. Thus, to confirm that the overproduction of MexAB-OprM was responsible for the reduced C-30 effect, we constructed *mexR*- and *nalD*-deficient *P. aeruginosa* (PRmexR2 and PRnalD1) using PAO1 as the parent strain. Deletion of *mexR* and *nalD* increased the expression of *mexAB-oprM* in *P. aeruginosa* (Table 2). In the presence of C-30, the colony counts of *P. aeruginosa* PRmexR2 and PRnalD1 treated with an NO donor were 4.1×10^3^ and 3.6×10^3^ c.f.u./100 µl (4.0 and 1.5 %), respectively, indicating that the *mexR*- and *nalD-*deficient *P. aeruginosa* are less sensitive to C-30 than *P. aeruginosa* PAO1 (Fig. 1c, d). Moreover, in the presence of both C-30 and PAβN, the colony counts of *P. aeruginosa* PRmexR2 and PRnalD1 were 2.7 and 1.5×10 c.f.u./100 µl (0.003 and 0.006 %), respectively, suggesting that the overproduction of MexAB-OprM was responsible for the reduced C-30 effect (Fig. 1c, d).

### Effect of QS–interfering agents on the sensitivity to nitrosative stress of *P. aeruginosa* clinical isolates

**Fig. 4.**
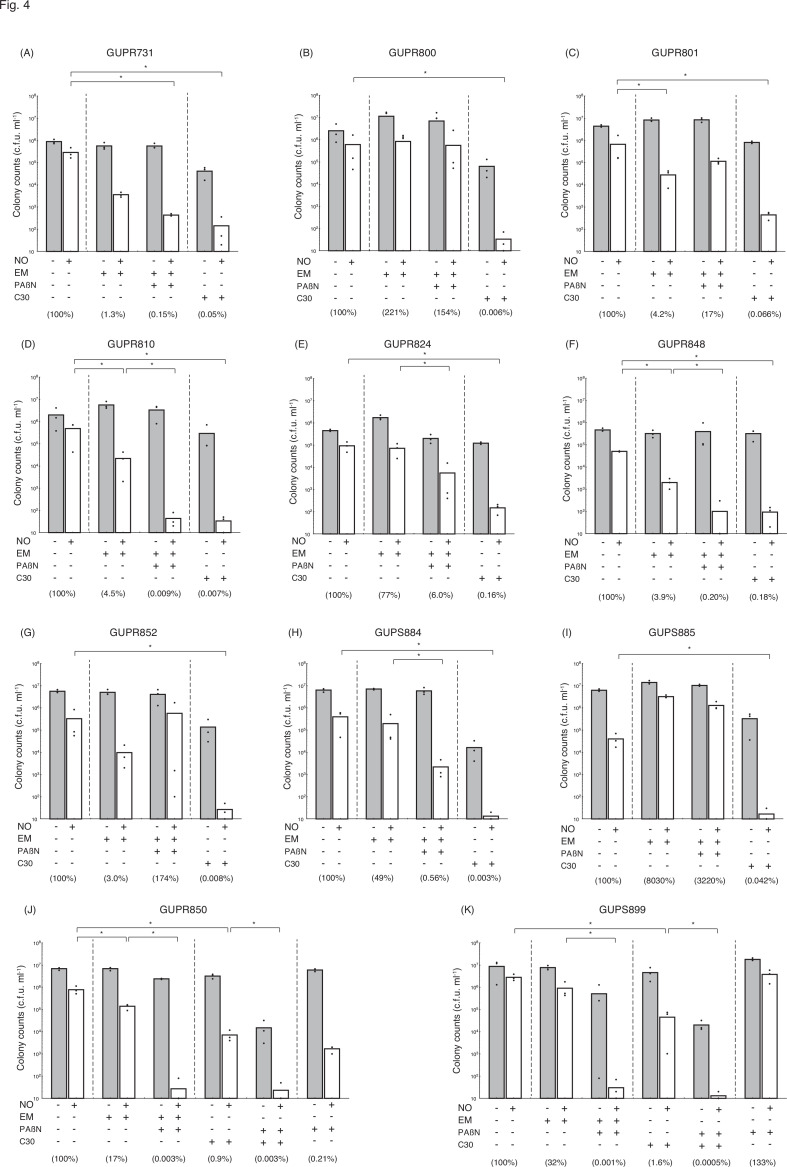
Effects of QS–interfering agents and EPI on the sensitivity to nitrosative stress of *P. aeruginosa* clinical isolates. Bacteria were grown in LB broth, with 10 µg ml^−1^ EM, 10 µM C-30, and/or 10 µg ml^−1^ PAβN as needed at 37 °C for 18 h. Bacteria were rinsed with saline solution containing 0.1 % glucose, 10 mM MOPS (pH 7.0), and the same concentration of EM, C-30, and/or PAβN. Bacterial suspensions were diluted 1 : 100 with saline solution containing 0.1 % glucose, 10 mM MOPS (pH 7.0), and the same concentration of EM, C-30, and/or PAβN with or without 100 µM DETA/NO, and then were incubated at 37 °C for 22 h. The number of viable bacteria was determined using bacteria plate counts. Percentages in parentheses represent the ratio to the colony count treated with the NO donor alone. All assays were performed at least three time. Results are expressed as the means and individual data points. Unpaired *t*-tests were used to determine significant differences when only two treatment groups were compared. One-way ANOVA with the Student–Newman–Keuls multiple comparisons test was used to analyse significant differences among multiple groups. Asterisks indicate a significant difference (*P<0.01). (**a**) GUPR731; (**b**) GUPR800; (**c**) GUPR801; (**d**) GUPR810; (**e**) GUPR824; (**f**) GUPR848; (**g**) GUPR852; (**h**) GUPS884; (**i**) GUPS885; (**j**) GUPR850; (**k**) GUPS899.

To investigate whether the increase in QS-interfering agent-dependent sensitivity to nitrosative stress is a typical characteristic of *P. aeruginosa*, we examined the viability of 11 clinical isolates of *P. aeruginosa* treated with NO donors. Although EM treatment was generally effective in reducing the viability of *P. aeruginosa* isolates treated with an NO donor, the effects of this were highly variable. This high variation in the EM augmentation of viability following treatment with a NO donor is notable because GUPR800, GUPR824, and GUPS884 were unaffected by EM, and treatment with EM in GUPS885 increased the viability, rather than inhibiting this (Fig. 4). Furthermore, high variability in the effects was also observed with treatment with both EM and PAβN, although this was generally effective in reducing the viability of *P. aeruginosa* isolates treated with an NO donor. In 7 out of 11 clinical isolates of *P. aeruginosa*, treatment with both EM and PAβN induced a loss of viability after treatment with an NO donor. GUPR800 and GUPS885 were unaffected by either EM or PAβN, and treatment with both EM and PAβN in GUPR801 and GUPR852 increased their viability, rather than inhibiting them (Fig. 4).

C-30 was also effective in reducing the viability of *P. aeruginosa* isolates treated with an NO donor. C-30 reduced the viability of the reference strain (PAO1) by 0.09 %, but on average, reduced the viability treated with an NO donor by only 0.27±0.15 % in the clinical isolates (Fig. 4). In 9 of 11 clinical isolates of *P. aeruginosa*, C-30 treatment alone significantly induced a loss of viability following treatment with an NO donor. In the remaining two *P. aeruginosa* strains (GUPR850 and GUPS899), treatment with C-30 did not reduce the viability of cells treated with an NO donor (Fig. 4), although treatment with both C-30 and PAβN was markedly more effective than treatment with C-30 alone. Thus, we confirmed the DNA sequences of genes regulating the expression of four types of efflux pumps and QS systems in 11 *P*. *aeruginosa* strains using whole-genome sequencing analysis data (accession number DRA009821). We found different frameshift mutations in *nalD* in GUPR850 and GUPS899, suggesting that these clinical isolates produced high levels of MexAB-OprM (Table S2).

## Discussion

This study demonstrated that QS–interfering agents, including macrolides, reduce the resistance of *P. aeruginosa* to host-defence mechanisms. Furthermore, multiple efflux pumps maintained by *P. aeruginosa* were found to be involved in suppressing the resistance-reducing effect of QS–interfering agents. These results indicate that the inhibition of the effect of EM on *P. aeruginosa* to reduce its virulence requires the production of MexAB-OprM and MexXY-OprM at normal levels and high production of MexCD-OprJ, and that suppression of the C-30 effect requires high production of MexAB-OprM, but not at normal levels.

This study used PAβN, the most well-characterized EPI [26,47, 48, 69], at low concentrations to clarify the role of the efflux pump in *P. aeruginosa*. PAβN broadly inhibits the function of multiple efflux pumps but at high concentrations can affect the permeability of the outer membrane of Gram-negative bacteria [70]. This effect was avoided in this study by the use of PAβN at 10 µg ml^−1^, which does not affect the outer membrane permeability of *P. aeruginosa* [70]. The specificity of the efflux pumps inhibited by PAβN was demonstrated in detail using mutants with only one efflux pump gene. PAβN efficiently inhibited the EM efflux capacity of MexAB-OprM produced at normal levels when the increase in NO sensitivity was indexed to the addition of low-dose EM. However, PAβN did not completely restore the EM efflux capacity of MexXY-OprM to normal levels. Furthermore, PAβN did not inhibit the EM efflux capacity of MexCD-OprJ at high levels, although this did inhibit the production of MexCD-OprJ at normal levels. Conversely, the results using PRmexR2, PRnalD1, and YM64 (pmexAB1) indicated that PAβN effectively inhibited the C-30 efflux capacity, even with the high expression of MexAB-OprM as indicated by the increased NO sensitivity induced by the addition of C-30. Therefore, the specificity of PAβN in *P. aeruginosa* effectively suppressed MexAB-OprM–mediated efflux but did not effectively suppress MexCD-OprJ– and MexXY-OprM–mediated efflux.

Several clinical isolates were unaffected by EM treatment alone in significantly reducing resistance to nitrosative stress. Furthermore, the effects of PAβN treatment in the presence of EM differed among these strains. The effectiveness of C-30 also varied in reducing nitrosative stress resistance. For example, EM treatment alone showed minimal effect on GUPR850 and GUPS899 but when combined with PAβN, the number of viable bacteria was significantly reduced. Furthermore, C-30 treatment alone in GUPR850 and GUPS899 was not as effective as in other strains; however, when combined with PAβN, this significantly reduced the number of viable bacteria. Conversely, GUPR800 was unaffected with both combined EM and PAβN treatments, as well as the EM treatment, whereas strains GUPR810 and GUPS884 were relatively affected by the combined EM and PAβN treatment. Such differences may be related to variations in the type and strength of active efflux pumps retained by the strains. The overproduction of MexAB-OprM and MexXY-OprM with mutations in *mexR*, *mexZ*, *nalC*, and *nalD* has been observed in clinical isolates that include epidemic strains [45,71,74]. Furthermore, quinolone-resistant *P. aeruginosa* isolates from CF lungs harboured mutations in *nfxB* [75]. Indeed, the decreased efficacy in treatment with C-30 in GUPR850 and GUPS899 compared with that in other strains can be explained by the presence of a frameshift mutation in *nalD*, which may produce strains with elevated MexAB-OprM production. In the remaining clinical isolates, one of probable factors contributing to the ineffectiveness of both the EM and PAβN treatments was the high production of MexCD-OprJ and/or MexXY-OprM.

*P. aeruginosa* porin OprD present in the outer membrane serves as a channel for the passage of basic amino acids, small peptides that contain these amino acids, and certain drugs, such as carbapenems, through the outer membrane [76,77]. We showed that macrolides were not involved in the passage through the outer membrane formed by OprD. However, *oprD*-deficient *P. aeruginosa* was susceptible to C-30, suggesting that OprD may play a role in reducing the concentration of C-30 in *P. aeruginosa*. Moreover, although *P. aeruginosa* strains are not susceptible to macrolides, a bulky antibiotic that is normally incapable of crossing the outer membrane of gram-negative bacteria, the overproduction of MexEF-OprN in *P. aeruginosa* increased the susceptibility to EM, probably because the structure of the outer membrane was changed by the overproduction of MexEF-OprN and EM permeability was increased. OprN penetrates the outer membrane; therefore, high OprN production may affect outer membrane permeability.

The mechanism of action of low-dose macrolide remains unclear. Macrolides inhibit the QS system, although the steps involved in this inhibition have not been fully revealed [7]. Macrolides are polyketide compounds characterized by the presence of 14-, 15-, or 16-membered aglycone ring lactone (e.g. EM, azithromycin, and josamycin, respectively) to which one or more amino and/or neutral sugars are attached [78]. Among these macrolides, 14-, 15-, and 16-membered aglycone ring lactone macrolides bind near the PTC loop with the 50S subunit of the bacterial ribosomal tunnel filled with water [79]. This is because classical antibacterial lactone macrolides have a 14–16 membered aglycone ring with hydrophobic and hydrophilic sides and relatively polar saccharide units [80,82]. However, only the 14- and 15-membered aglycone ring macrolides are effective against *P. aeruginosa* cells [62,83, 84]. Therefore, the 14- and 15-membered aglycone ring structures of macrolides may be important for inhibiting the QS system. C-30 is a QS-interfering agent that antagonistically inhibits the binding of lasR and AHL [24,25]. Thus, QS-interfering agents such as EM and C-30 are effective in treating *P. aeruginosa* infections because they reduce the virulence of *P. aeruginosa*, and consequently long-term low-dose macrolide therapy is widely used as standard treatment [85]. During NO^3-^ reduction to N_2_ in denitrification, NO^3-^, NO^2-^, NO, and N_2_O reductases are involved. In the *rhlR* mutant of *P. aeruginosa,* NO^3-^ and NO^2-^ reductases activities are upregulated five and sevenfold, respectively [86]. Moreover, the lack of *rhlR* forces bacteria to undergo metabolic suicide by NO overproduction [86]. Thus, since QS regulates the denitrification rate [86,88], QS dysregulation by QS–interfering agents may increase the susceptibility of *P. aeruginosa* to NO.

C-30 was more effective than EM in increasing susceptibility to NO (Fig. S2), and was effective against most *P. aeruginosa* clinical isolates (Fig. 4). Although several *P. aeruginosa* strains ineffective against macrolides have been reported [62], resistance to QS–interfering agents such as C-30 has not been easily observed. However, C-30 resistance in *P. aeruginosa* has been demonstrated in the laboratory [89]. Two clinical isolates (GUPR850 and GUPS899) were insufficiently resistant to C-30 in this study. Therefore, the combination of EM and PAβN, or C-30 and PAβN could be highly effective as a treatment for *P. aeruginosa* infections. Since efflux pumps are not essential for bacterial survival, the development of bacterial EPI-resistance may be difficult. However, the toxicity of existing EPI compounds hinders their clinical application. In particular, PAβN has been found to be toxic to eukaryotic cells [49,90, 91]. Research is underway to reduce the toxicity of the PAβN-compound series [92,93], and the structure–activity relationship of these compounds showed that other PAβN derivatives are more active with higher solubility in biological fluids and lower toxicity levels, and to develop other structurally different types of EPI [50,51, 94, 95].

## supplementary material

10.1099/mic.0.001464 Supplementary Material 1.

## Abbreviations

AHLs: acyl homoserine lactones
C-30: furanone C-30
CF: cystic fibrosis
DETA/NO: DETA NONOate
DPB: diffuse panbronchiolitis
EM: erythromycin
EPI: efflux pump inhibitor
MDRP: multidrug-resistant P. aeruginosa
NO: nitric oxide
PAβN: phenylalanine arginyl β-naphthylamide
PQS: Pseudomonas quinolone signal
QS: quorum sensing
RND: resistance/nodulation/division

## References

[1] Høiby N (1994). Diffuse panbronchiolitis and cystic fibrosis: East meets West. Thorax.

[2] Schultz MJ (2004). Macrolide activities beyond their antimicrobial effects: macrolides in diffuse panbronchiolitis and cystic fibrosis. J Antimicrob Chemother.

[3] Fujii T, Kadota J, Kawakami K, Iida K, Shirai R (1995). Long term effect of erythromycin therapy in patients with chronic *Pseudomonas aeruginosa* infection. Thorax.

[4] Jaffé A, Francis J, Rosenthal M, Bush A (1998). Long-term azithromycin may improve lung function in children with cystic fibrosis. Lancet.

[5] Bulska M, Orszulak-Michalak D (2014). Immunomodulatory and anti-inflammatory properties of macrolides. Curr Issues Pharm Med Sci.

[6] Imperi F, Leoni L, Visca P (2014). Antivirulence activity of azithromycin in *Pseudomonas aeruginosa*. Front Microbiol.

[7] Leroy A-G, Caillon J, Caroff N, Broquet A, Corvec S (2021). Could azithromycin be part of *Pseudomonas aeruginosa* acute pneumonia treatment?. Front Microbiol.

[8] Tateda K, Comte R, Pechere JC, Köhler T, Yamaguchi K (2001). Azithromycin inhibits quorum sensing in *Pseudomonas aeruginosa*. Antimicrob Agents Chemother.

[9] Wagner T, Soong G, Sokol S, Saiman L, Prince A (2005). Effects of azithromycin on clinical isolates of *Pseudomonas aeruginosa* from cystic fibrosis patients. Chest.

[10] Wagner VE, Bushnell D, Passador L, Brooks AI, Iglewski BH (2003). Microarray analysis of *Pseudomonas aeruginosa* quorum-sensing regulons: effects of growth phase and environment. J Bacteriol.

[11] Nalca Y, Jänsch L, Bredenbruch F, Geffers R, Buer J (2006). Quorum-sensing antagonistic activities of azithromycin in *Pseudomonas aeruginosa* PAO1: a global approach. Antimicrob Agents Chemother.

[12] Zeng J, Zhang N, Huang B, Cai R, Wu B (2016). Mechanism of azithromycin inhibition of HSL synthesis in *Pseudomonas aeruginosa*. Sci Rep.

[13] Skindersoe ME, Alhede M, Phipps R, Yang L, Jensen PO (2008). Effects of antibiotics on quorum sensing in *Pseudomonas aeruginosa*. Antimicrob Agents Chemother.

[14] Fuqua C, Parsek MR, Greenberg EP (2001). Regulation of gene expression by cell-to-cell communication: acyl-homoserine lactone quorum sensing. Annu Rev Genet.

[15] Pearson JP, Gray KM, Passador L, Tucker KD, Eberhard A (1994). Structure of the autoinducer required for expression of *Pseudomonas aeruginosa* virulence genes. Proc Natl Acad Sci U S A.

[16] Ochsner UA, Reiser J (1995). Autoinducer-mediated regulation of rhamnolipid biosurfactant synthesis in *Pseudomonas aeruginosa*. Proc Natl Acad Sci U S A.

[17] Gambello MJ, Iglewski BH (1991). Cloning and characterization of the *Pseudomonas aeruginosa* lasR gene, a transcriptional activator of elastase expression. J Bacteriol.

[18] Pesci EC, Milbank JB, Pearson JP, McKnight S, Kende AS (1999). Quinolone signaling in the cell-to-cell communication system of *Pseudomonas aeruginosa*. Proc Natl Acad Sci U S A.

[19] Pesci EC, Pearson JP, Seed PC, Iglewski BH (1997). Regulation of las and rhl quorum sensing in *Pseudomonas aeruginosa*. J Bacteriol.

[20] Rasko DA, Sperandio V (2010). Anti-virulence strategies to combat bacteria-mediated disease. Nat Rev Drug Discov.

[21] Ding X, Yin B, Qian L, Zeng Z, Yang Z (2011). Screening for novel quorum-sensing inhibitors to interfere with the formation of *Pseudomonas aeruginosa* biofilm. J Med Microbiol.

[22] Rasmussen TB, Bjarnsholt T, Skindersoe ME, Hentzer M, Kristoffersen P (2005). Screening for quorum-sensing inhibitors (QSI) by use of a novel genetic system, the QSI selector. J Bacteriol.

[23] Hentzer M, Wu H, Andersen JB, Riedel K, Rasmussen TB (2003). Attenuation of *Pseudomonas aeruginosa* virulence by quorum sensing inhibitors. EMBO J.

[24] Kim C, Kim J, Park H-Y, Park H-J, Lee JH (2008). Furanone derivatives as quorum-sensing antagonists of *Pseudomonas aeruginosa*. Appl Microbiol Biotechnol.

[25] Yang L, Rybtke MT, Jakobsen TH, Hentzer M, Bjarnsholt T (2009). Computer-aided identification of recognized drugs as *Pseudomonas aeruginosa* quorum-sensing inhibitors. Antimicrob Agents Chemother.

[26] Askoura M, Mottawea W, Abujamel T, Taher I (2011). Efflux pump inhibitors (EPIs) as new antimicrobial agents against *Pseudomonas aeruginosa*. Libyan J Med.

[27] Lister PD, Wolter DJ, Hanson ND (2009). Antibacterial-resistant *Pseudomonas aeruginosa*: clinical impact and complex regulation of chromosomally encoded resistance mechanisms. Clin Microbiol Rev.

[28] Piddock LJV (2006). Clinically relevant chromosomally encoded multidrug resistance efflux pumps in bacteria. Clin Microbiol Rev.

[29] Li X-Z, Plésiat P, Nikaido H (2015). The challenge of efflux-mediated antibiotic resistance in Gram-negative bacteria. Clin Microbiol Rev.

[30] Li XZ, Nikaido H, Poole K (1995). Role of mexA-mexB-oprM in antibiotic efflux in *Pseudomonas aeruginosa*. Antimicrob Agents Chemother.

[31] Li XZ, Barré N, Poole K (2000). Influence of the MexA-MexB-oprM multidrug efflux system on expression of the MexC-MexD-oprJ and MexE-MexF-oprN multidrug efflux systems in *Pseudomonas aeruginosa*. J Antimicrob Chemother.

[32] Evans K, Adewoye L, Poole K (2001). MexR repressor of the mexAB-oprM multidrug efflux operon of *Pseudomonas aeruginosa*: identification of MexR binding sites in the mexA-mexR intergenic region. J Bacteriol.

[33] Cao L, Srikumar R, Poole K (2004). MexAB-OprM hyperexpression in NalC-type multidrug-resistant *Pseudomonas aeruginosa*: identification and characterization of the nalC gene encoding a repressor of PA3720-PA3719. Mol Microbiol.

[34] Sobel ML, Hocquet D, Cao L, Plesiat P, Poole K (2005). Mutations in PA3574 (nalD) lead to increased MexAB-OprM expression and multidrug resistance in laboratory and clinical isolates of *Pseudomonas aeruginosa*. Antimicrob Agents Chemother.

[35] Daigle DM, Cao L, Fraud S, Wilke MS, Pacey A (2007). Protein modulator of multidrug efflux gene expression in *Pseudomonas aeruginosa*. J Bacteriol.

[36] Starr LM, Fruci M, Poole K (2012). Pentachlorophenol induction of the *Pseudomonas aeruginosa* mexAB-oprM efflux operon: involvement of repressors NalC and MexR and the antirepressor ArmR. PLoS One.

[37] Morita Y, Cao L, Gould VC, Avison MB, Poole K (2006). nalD encodes a second repressor of the mexAB-oprM multidrug efflux operon of *Pseudomonas aeruginosa*. J Bacteriol.

[38] Srikumar R, Tsang E, Poole K (1999). Contribution of the MexAB-OprM multidrug efflux system to the beta-lactam resistance of penicillin-binding protein and beta-lactamase-derepressed mutants of *Pseudomonas aeruginosa*. J Antimicrob Chemother.

[39] Okamoto K, Gotoh N, Nishino T (2002). Alterations of susceptibility of *Pseudomonas aeruginosa* by overproduction of multidrug efflux systems, MexAB-OprM, MexCD-OprJ, and MexXY/OprM to carbapenems: substrate specificities of the efflux systems. J Infect Chemother.

[40] Llanes C, Köhler T, Patry I, Dehecq B, van Delden C (2011). Role of the MexEF-OprN efflux system in low-level resistance of *Pseudomonas aeruginosa* to ciprofloxacin. Antimicrob Agents Chemother.

[41] Hocquet D, Vogne C, El Garch F, Vejux A, Gotoh N (2003). MexXY-OprM efflux pump is necessary for a adaptive resistance of *Pseudomonas aeruginosa* to aminoglycosides. Antimicrob Agents Chemother.

[42] Masuda N, Sakagawa E, Ohya S, Gotoh N, Tsujimoto H (2000). Contribution of the MexX-MexY-oprM efflux system to intrinsic resistance in *Pseudomonas aeruginosa*. Antimicrob Agents Chemother.

[43] Morita Y, Tomida J, Kawamura Y (2012). MexXY multidrug efflux system of *Pseudomonas aeruginosa*. Front Microbiol.

[44] Cabot G, Ocampo-Sosa AA, Tubau F, Macia MD, Rodríguez C (2011). Overexpression of AmpC and efflux pumps in *Pseudomonas aeruginosa* isolates from bloodstream infections: prevalence and impact on resistance in a Spanish multicenter study. Antimicrob Agents Chemother.

[45] Llanes C, Hocquet D, Vogne C, Benali-Baitich D, Neuwirth C (2004). Clinical strains of Pseudomonas aeruginosa overproducing MexAB-OprM and MexXY efflux pumps simultaneously. Antimicrob Agents Chemother.

[46] Shigemura K, Osawa K, Kato A, Tokimatsu I, Arakawa S (2015). Association of overexpression of efflux pump genes with antibiotic resistance in *Pseudomonas aeruginosa* strains clinically isolated from urinary tract infection patients. J Antibiot.

[47] Pagès J-M, Masi M, Barbe J (2005). Inhibitors of efflux pumps in Gram-negative bacteria. Trends Mol Med.

[48] Dreier J, Ruggerone P (2015). Interaction of antibacterial compounds with RND eﬄux pumps in *Pseudomonas aeruginosa*. Front Microbiol.

[49] Opperman TJ, Nguyen ST (2015). Recent advances toward a molecular mechanism of efflux pump inhibition. Front Microbiol.

[50] Aron Z, Opperman TJ (2016). Optimization of a novel series of pyranopyridine RND efflux pump inhibitors. Curr Opin Microbiol.

[51] Compagne N, Vieira Da Cruz A, Müller RT, Hartkoorn RC, Flipo M (2023). Update on the discovery of efflux pump inhibitors against critical priority Gram-negative bacteria. Antibiotics.

[52] Lomovskaya O, Warren MS, Lee A, Galazzo J, Fronko R (2001). Identification and characterization of inhibitors of multidrug resistance efflux pumps in *Pseudomonas aeruginosa*: novel agents for combination therapy. Antimicrob Agents Chemother.

[53] MacMicking J, Xie QW, Nathan C (1997). Nitric oxide and macrophage function. Annu Rev Immunol.

[54] Bogdan C (2001). Nitric oxide and the immune response. Nat Immunol.

[55] Goretski J, Zafiriou OC, Hollocher TC (1990). Steady-state nitric oxide concentrations during denitrification. J Biol Chem.

[56] Watmough NJ, Butland G, Cheesman MR, Moir JW, Richardson DJ (1999). Nitric oxide in bacteria: synthesis and consumption. Biochim Biophys Acta.

[57] Sobko T, Reinders C, Norin E, Midtvedt T, Gustafsson LE (2004). Gastrointestinal nitric oxide generation in germ-free and conventional rats. Am J Physiol Gastrointest Liver Physiol.

[58] Chin MP, Schauer DB, Deen WM (2008). Prediction of nitric oxide concentrations in colonic crypts during inflammation. Nitric Oxide.

[59] Elphick HE, Demoncheaux EA, Ritson S, Higenbottam TW, Everard ML (2001). Exhaled nitric oxide is reduced in infants with cystic fibrosis. Thorax.

[60] Jöbsis Q, Raatgeep HC, Schellekens SL, Kroesbergen A, Hop WC (2000). Hydrogen peroxide and nitric oxide in exhaled air of children with cystic fibrosis during antibiotic treatment. Eur Respir J.

[61] Mhanna MJ, Ferkol T, Martin RJ, Dreshaj IA, van Heeckeren AM (2001). Nitric oxide deficiency contributes to impairment of airway relaxation in cystic fibrosis mice. Am J Respir Cell Mol Biol.

[62] Shimizu T, Miyoshi-Akiyama T, Ogura K, Murata S, Ishige S (2020). Effect of sub-MICs of macrolides on the sensitivity of *Pseudomonas aeruginosa* to nitrosative stress: effectiveness against *P. aeruginosa* with and without multidrug resistance. Antimicrob Agents Chemother.

[63] Nozaki S, Niki H (2019). Exonuclease III (XthA) enforces *In Vivo* DNA cloning of *Escherichia coli* to create cohesive ends. J Bacteriol.

[64] Dumas J-L, van Delden C, Perron K, Köhler T (2006). Analysis of antibiotic resistance gene expression in *Pseudomonas aeruginosa* by quantitative real-time-PCR. FEMS Microbiol Lett.

[65] Purssell A, Poole K (2013). Functional characterization of the NfxB repressor of the mexCD-oprJ multidrug efflux operon of *Pseudomonas aeruginosa*. Microbiology.

[66] Lau CH-F, Fraud S, Jones M, Peterson SN, Poole K (2012). Reduced expression of the rplU-rpmA ribosomal protein operon in mexXY-expressing pan-aminoglycoside-resistant mutants of *Pseudomonas aeruginosa*. Antimicrob Agents Chemother.

[67] Livak KJ, Schmittgen TD (2001). Analysis of relative gene expression data using real-time quantitative PCR and the 2(-Delta Delta C(T)) Method. Methods.

[68] Morita Y, Komori Y, Mima T, Kuroda T, Mizushima T (2001). Construction of a series of mutants lacking all of the four major mex operons for multidrug efflux pumps or possessing each one of the operons from *Pseudomonas aeruginosa* PAO1: MexCD-OprJ is an inducible pump. FEMS Microbiol Lett.

[69] Renau TE, Léger R, Flamme EM, Sangalang J, She MW (1999). Inhibitors of efflux pumps in *Pseudomonas aeruginosa* potentiate the activity of the fluoroquinolone antibacterial levofloxacin. J Med Chem.

[70] Lamers RP, Cavallari JF, Burrows LL (2013). The efflux inhibitor phenylalanine-arginine beta-naphthylamide (PAβN) permeabilizes the outer membrane of gram-negative bacteria. PLoS One.

[71] Quale J, Bratu S, Gupta J, Landman D (2006). Interplay of efflux system, ampC, and oprD expression in carbapenem resistance of *Pseudomonas aeruginosa* clinical isolates. Antimicrob Agents Chemother.

[72] Tomás M, Doumith M, Warner M, Turton JF, Beceiro A (2010). Efflux pumps, OprD porin, AmpC beta-lactamase, and multiresistance in *Pseudomonas aeruginosa* isolates from cystic fibrosis patients. Antimicrob Agents Chemother.

[73] Sobel ML, Neshat S, Poole K (2005). Mutations in PA2491 (mexS) promote MexT-dependent mexEF-oprN expression and multidrug resistance in a clinical strain of *Pseudomonas aeruginosa*. J Bacteriol.

[74] Hocquet D, Nordmann P, El Garch F, Cabanne L, Plésiat P (2006). Involvement of the MexXY-OprM efflux system in emergence of cefepime resistance in clinical strains of *Pseudomonas aeruginosa*. Antimicrob Agents Chemother.

[75] Jalal S, Ciofu O, Hoiby N, Gotoh N, Wretlind B (2000). Molecular mechanisms of fluoroquinolone resistance in *Pseudomonas aeruginosa* isolates from cystic fibrosis patients. Antimicrob Agents Chemother.

[76] Trias J, Nikaido H (1990). Outer membrane protein D2 catalyzes facilitated diffusion of carbapenems and penems through the outer membrane of *Pseudomonas aeruginosa*. Antimicrob Agents Chemother.

[77] Trias J, Nikaido H (1990). Protein D2 channel of the *Pseudomonas aeruginosa* outer membrane has a binding site for basic amino acids and peptides. J Biol Chem.

[78] Fyfe C, Grossman TH, Kerstein K, Sutcliffe J (2016). Resistance to macrolide antibiotics in Public Health Pathogens. Cold Spring Harb Perspect Med.

[79] Janas A, Przybylski P (2019). 14- and 15-membered lactone macrolides and their analogues and hybrids: structure, molecular mechanism of action and biological activity. Eur J Med Chem.

[80] Tenson T, Lovmar M, Ehrenberg M (2003). The mechanism of action of macrolides, lincosamides and streptogramin B reveals the nascent peptide exit path in the ribosome. J Mol Biol.

[81] Yonath A (2005). Antibiotics targeting ribosomes: resistance, selectivity, synergism and cellular regulation. Annu Rev Biochem.

[82] Arsic B, Awan A, Brennan RJ, Aguilar JA, Ledder R (2014). Theoretical and experimental investigation on clarithromycin, erythromycin A and azithromycin and descladinosyl derivatives of clarithromycin and azithromycin with 3-O substitution as anti-bacterial agents. Med Chem Commun.

[83] Tateda K, Hirakata Y, Furuya N, Ohno A, Yamaguchi K (1993). Effects of sub-MICs of erythromycin and other macrolide antibiotics on serum sensitivity of *Pseudomonas aeruginosa*. Antimicrob Agents Chemother.

[84] Tateda K, Ishii Y, Matsumoto T, Furuya N, Nagashima M (1996). Direct evidence for antipseudomonal activity of macrolides: exposure-dependent bactericidal activity and inhibition of protein synthesis by erythromycin, clarithromycin, and azithromycin. Antimicrob Agents Chemother.

[85] Chalmers JD (2017). Macrolide resistance in *Pseudomonas aeruginosa*: implications for practice. Eur Respir J.

[86] Yoon SS, Hennigan RF, Hilliard GM, Ochsner UA, Parvatiyar K (2002). *Pseudomonas aeruginosa* anaerobic respiration in biofilms: relationships to cystic fibrosis pathogenesis. Dev Cell.

[87] Hassett DJ, Cuppoletti J, Trapnell B, Lymar SV, Rowe JJ (2002). Anaerobic metabolism and quorum sensing by *Pseudomonas aeruginosa* biofilms in chronically infected cystic fibrosis airways: rethinking antibiotic treatment strategies and drug targets. Adv Drug Deliv Rev.

[88] Toyofuku M, Nomura N, Fujii T, Takaya N, Maseda H (2007). Quorum sensing regulates denitrification in *Pseudomonas aeruginosa* PAO1. J Bacteriol.

[89] Maeda T, García-Contreras R, Pu M, Sheng L, Garcia LR (2012). Quorum quenching quandary: resistance to antivirulence compounds. ISME J.

[90] Lomovskaya O, Bostian KA (2006). Practical applications and feasibility of efflux pump inhibitors in the clinic--a vision for applied use. Biochem Pharmacol.

[91] Venter H, Mowla R, Ohene-Agyei T, Ma S (2015). RND-type drug eﬄux pumps from Gram-negative bacteria: molecular mechanism and inhibition. Front Microbiol.

[92] Renau TE, Léger R, Flamme EM, Sangalang J, She MW (1999). Inhibitors of efflux pumps in Pseudomonas aeruginosa potentiate the activity of the fluoroquinolone antibacterial levofloxacin. J Med Chem.

[93] Watkins WJ, Landaverry Y, Léger R, Litman R, Renau TE (2003). The relationship between physicochemical properties, in vitro activity and pharmacokinetic profiles of analogues of diamine-containing efflux pump inhibitors. Bioorg Med Chem Lett.

[94] Plé C, Tam H-K, Vieira Da Cruz A, Compagne N, Jiménez-Castellanos J-C (2022). Pyridylpiperazine-based allosteric inhibitors of RND-type multidrug efflux pumps. Nat Commun.

[95] Zhang Y, Rosado-Lugo JD, Datta P, Sun Y, Cao Y (2022). Evaluation of a conformationally constrained indole carboxamide as a potential efflux pump inhibitor in *Pseudomonas aeruginosa*. Antibiotics (Basel).

[96] Stover CK, Pham XQ, Erwin AL, Mizoguchi SD, Warrener P (2000). Complete genome sequence of *Pseudomonas aeruginosa* PAO1, an opportunistic pathogen. Nature.

[97] Simon R, Priefer U, Pühler A (1983). A broad host range mobilization system for In Vivo genetic engineering: transposon mutagenesis in gram negative bacteria. Nat Biotechnol.

[98] West SE, Schweizer HP, Dall C, Sample AK, Runyen-Janecky LJ (1994). Construction of improved Escherichia-Pseudomonas shuttle vectors derived from pUC18/19 and sequence of the region required for their replication in Pseudomonas aeruginosa. Gene.

[99] Hoang TT, Karkhoff-Schweizer RR, Kutchma AJ, Schweizer HP (1998). A broad-host-range Flp-FRT recombination system for site-specific excision of chromosomally-located DNA sequences: application for isolation of unmarked *Pseudomonas aeruginosa* mutants. Gene.

