# Supplementary material for: Effects of quorum sensing–interfering agents, including macrolides and furanone C-30, and an efflux pump inhibitor on nitrosative stress sensitivity in Pseudomonas aeruginosa
Source: Microbiology (Reading). 2024 Jun 20;170(6):001464. doi: 10.1099/mic.0.001464 (PMC11263931; doi:10.1099/mic.0.001464)
Supplement: Supplementary Material 1. [file mic-170-01464-s001.pdf]

Supplemental Table S1 PCR Primers

Primer	Sequence
P1394	GAGCTCGAATTAGCTTCA
P1395	TCGAGCTTAACCCCTAG
P1396	GGTACCGAGCTCGAATT
P1397	CTGCAGGCATGCAAGCT
P1431	GACCATGATTACGAATTCGAGCTCGGTACCGGTTCCGCGCCAGCTCA
P1433	TAGGAACTTCAAGATCCCCAATTCGAGCTCCCCTCGCGCCCGCGGCG
P1434	ACGGCCAGTGCCAAGCTTGCATGCCTGCAGGGGAGCAGGGCGAGGGC
P1435	TCAGAGCGCTTTTGAAGCTAATTCGAGCTCCGATGGGTCCCGGTTGG
P1438	GACCATGATTACGAATTCGAGCTCGGTACCTGAGGATGATGCCGTTC
P1439	ACGGCCAGTGCCAAGCTTGCATGCCTGCAGCTGTACGGCCGCTTCAA
P1446	TCAGAGCGCTTTTGAAGCTAATTCGAGCTCTGGTTTGGCCGAGTAAAC
P1447	TAGGAACTTCAAGATCCCCAATTCGAGCTCGAACATTCTTTTCGAAGCA
P1452	CGACCAGGCCGTGAGCAAGCAGC
P1453	GGAGACCTTCGCCGCGTTGTCGC
P1456	CCAGGACCAGCACGAACTTCTTGC
P1457	CGACAACGCCAAGGGCGAGTTCACC
P1460	GCAAGCGCATGGTCGACAAGA
P1461	CGCTGTGCTCTTGCAGGTTGTGA
P1462	CTATCGGCATCACCAGCG
P1463	ATCTGGAACAGCACGGTG
P1464	CTCGAGCTATACGTGCCTAAC
P1465	GTCCCTCTTCCCATTTCACG
P1482	GACCATGATTACGAATTCGAGCTCGGTACCCAATCAACGGTCGGGTGTGTCATGGCTGAT
P1483	ACGGCCAGTGCCAAGCTTGCATGCCTGCAGCACCCGCTAGCACCGTTTCCCACACGTTTA
P1484	GACCATGATTACGAATTCGAGCTCGGTACCAATCCCGAAAAACCGACTGGCGGAGTCAAG
P1485	ACGGCCAGTGCCAAGCTTGCATGCCTGCAGGAGTGGCCGATTTCCATCGGCCGGGGGATA
P1486	GACCATGATTACGAATTCGAGCTCGGTACCGCTTTCGCTCATGAGGACAACGCTATGCAA
P1487	ACGGCCAGTGCCAAGCTTGCATGCCTGCAGCTAGGGTCGGCGTTCTTGCATGGCGCGGAA
P1488	GACCATGATTACGAATTCGAGCTCGGTACCAAGCTCGCGAGTTCACGAATCGAGGGACAC
P1489	ATCAGGCTTGCTCCGTGGGGATCTGGGGCG
P1490	CGCCCCAGATCCCCACGGAGCAAGCCTGATATGAAACGGTCCTTCCTTTCCCTGGCGGTA
P1565	GACCATGATTACGAATTCGAGCTCGGTACCCAAACGCATTCGCCACA
P1566	TCAGAGCGCTTTTGAAGCTAATTCGAGCTCTGTGATTGCTCCTTTGGT
P1567	TAGGAACTTCAAGATCCCCAATTCGAGCTCTCGACCGACAGGCAACG
P1568	ACGGCCAGTGCCAAGCTTGCATGCCTGCAGGGCCATCGATGATGAGG
P1636	GACCATGATTACGAATTCGAGCTCGGTACCCCAGGCTCAGCTGGACG
P1637	TCAGAGCGCTTTTGAAGCTAATTCGAGCTCGCTGTGCTGGGTAGTCG
P1638	TAGGAACTTCAAGATCCCCAATTCGAGCTCCCCTCCTCGCGGATCGC
P1639	ACGGCCAGTGCCAAGCTTGCATGCCTGCAGGCAGGGTGAAGACGTTG

Supplemental Table S2 Amino acid mutations of efflux pump regulators and quorum sensing receptors in clinical isolates of *P. aeruginosa*

Strains	*mexAB-oprM*				*mexCD-oprJ*		*mexEF-oprN*		*mexXY-oprM*		quorum sensing receptors		
	mexR	*nalD*	*nalC*	*armR*	*nfxB*	*esrC*	*mexT*	*mexS*	*mexZ (amrR)*	*armZ*	*lasR*	*rhlR*	*mvfR (pqsR)*
	PA0424	PA3574	PA3721	PA3719	PA4600	PA4596	PA2492	PA2491	PA2020	PA5471	PA1430	PA3477	PA1003
GUPR731		Trp49Ser	Gly71Glu<br>Gln182Lys<br>Leu206Val<br>Ser209Arg	Thr5Ala<br>Ser21Thr	His109Tyr	Asn3Ser<br>Glu61Asp<br>Arg88His<br>Val101Ile	Glu26Gly<br>Pro60Ser<br>Phe172Ile	Lys17Thr<br>Ala175Val<br>Asp249Asn<br>Ser289Thr<br>Val308Ile<br>Val318Ile	Ser116fs<br>Leu196Ile	AlaLeu87ValPro<br>Asp161Gly<br>His182Gln<br>Val243Ala<br>Glu307Asp<br>Ile346Val	Trp152*		Ala34Val<br>Ile310Leu<br>Ala314Val
GUPR800	Val126Glu	Val151_Gly156del	Gly71Glu<br>Glu153Gln<br>Ser209Arg	Ser21Thr<br>Gly23Glu<br>Tyr32Cys	Gly129*		Pro60Ser<br>Gln80fs<br>Phe172Ile	Asp249Asn	Ala194Asp	Leu88Pro<br>Asp161Gly<br>His182Gln<br>Arg204Cys<br>Val243Ala	Ala50_Asn55del		Gln14*<br>Ala314Val
GUPR801	Val126Glu		Gly71Glu<br>Glu153Gln<br>Ser209Arg	Ser21Thr<br>Gly23Glu<br>Tyr32Cys			Gln80fs<br>Phe172Ile	Asp249Asn	Glu21_Lys27del	Leu88Pro<br>Asp161Gly<br>His182Gln<br>Arg204Cys<br>Val243Ala			Ala314Val
GUPR810	Gly51Arg,<br>Val126Glu		Gly71Glu<br>Ser209Arg	Ser21Thr<br>Tyr32Cys		Gln89His	Gln80fs<br>Phe172Ile	Asp249Asn	Leu138Arg	Cys40Arg<br>Leu88Pro<br>Ser112Asn<br>Asp119Glu<br>Asp207Asn<br>Ile237Val<br>Val243Ala<br>Ser314Asn			
GUPR824		Thr158Pro	Gly71Glu				Pro60Ser<br>Gln80fs<br>Phe172Ile	Asp249Asn		Leu88Pro<br>Asp161Gly<br>Leu174Phe<br>His182Gln<br>Val243Ala			
GUPR848	Val126Glu		Gly71Glu<br>Glu153Gln<br>Ser209Arg	Ser21Thr<br>Gly23Glu<br>Tyr32Cys			Glu26Gly<br>Pro60Ser<br>Phe172Ile	Asp249Asn	Glu98fs	Leu88Pro<br>Asp161Gly<br>His182Gln<br>Arg204Cys<br>Val243Ala			Ala314Val
GUPR850	Val126Glu	Asn175fs	Gly71Glu<br>Ser209Arg	Ser21Thr		Arg35Gln	Gln80fs<br>Phe172Ile	Val194Glu<br>Asp249Asn		Leu88Pro<br>Asp161Gly<br>His182Gln<br>Arg204Cys<br>Val243Ala	Cys79Tyr	His183fs	Ala314Val
GUPR852	Val126Glu	Val151_Gly156del	Gly71Glu<br>Glu153Gln<br>Ser209Arg	Ser21Thr<br>Gly23Glu<br>Tyr32Cys			Pro60Ser<br>Gln80fs<br>Phe172Ile	Asp249Asn	Ala194Asp	Leu88Pro<br>Asp161Gly<br>His182Gln<br>Arg204Cys<br>Val243Ala	Ala50_Asn55del		Gln14*<br>Ala314Val
GUPS884	Ala103Gly,<br>Val126Glu			Thr5Ala<br>Ser21Thr	His109Tyr	Asn3Ser<br>Glu61Asp<br>Arg88His<br>Val101Ile	Glu26Gly<br>Pro60Ser<br>Gln80fs<br>Phe172Ile	Lys17Thr<br>Ala175Val<br>Glu181Asp<br>Asp249Asn<br>Ser289Thr<br>Val308Ile<br>Val318Ile	Leu138Arg<br>Glu189_Ala194del<br>Leu196Ile	Cys40Arg<br>AlaLeu87ValPro<br>Ala128Glu<br>Asp161Gly<br>His182Gln<br>Val243Ala<br>Glu307Asp<br>Ile346Val	Ala227Val		Ile310Leu<br>Ala314Val
GUPS885	Thr130Pro						Pro60Ser<br>Gln80fs<br>Phe172Ile	Ala75Val<br>Asp249Asn	Gly15fs	Leu88Pro<br>Gly157Asp<br>Asp161Gly<br>His182Gln<br>Val243Ala			
GUPS899	Val126Glu	Val133fs	Gly71Glu<br>Ser209Arg		Ala141_Ala142dup		Gln80fs<br>Phe172Ile	Asp249Asn		Leu88Pro<br>Asp159Asn<br>Asp161Gly<br>His182Gln<br>Val243Ala			Ala314Val

P. aeruginosa PAO1 was used as reference strain.
fs: frameshift; *: nonsense mutation; dup: duplication; del: deletion

Supplemental Figure 1

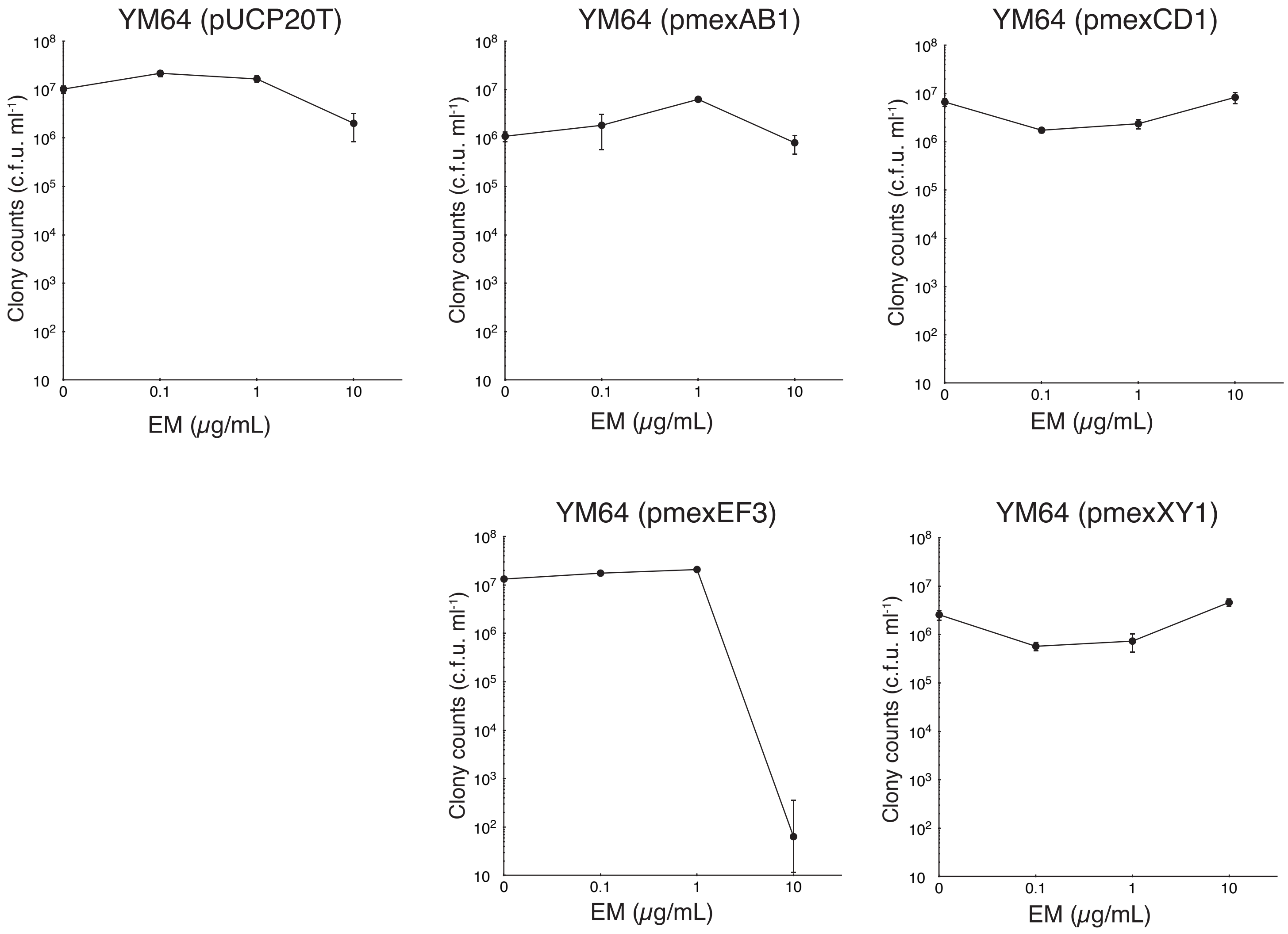

Fig. S1

Effect of EM on growth of efflux pump gene-overexpressed *P. aeruginosa*. Bacteria were grown in LB broth containing various concentrations of EM at 37°C for 18 h. The number of bacteria was determined using bacteria plate counts (cfu). All assays were independently repeated three times. Results are expressed as the means ± standard errors for the triplicate experiments.

Supplemental Figure 2

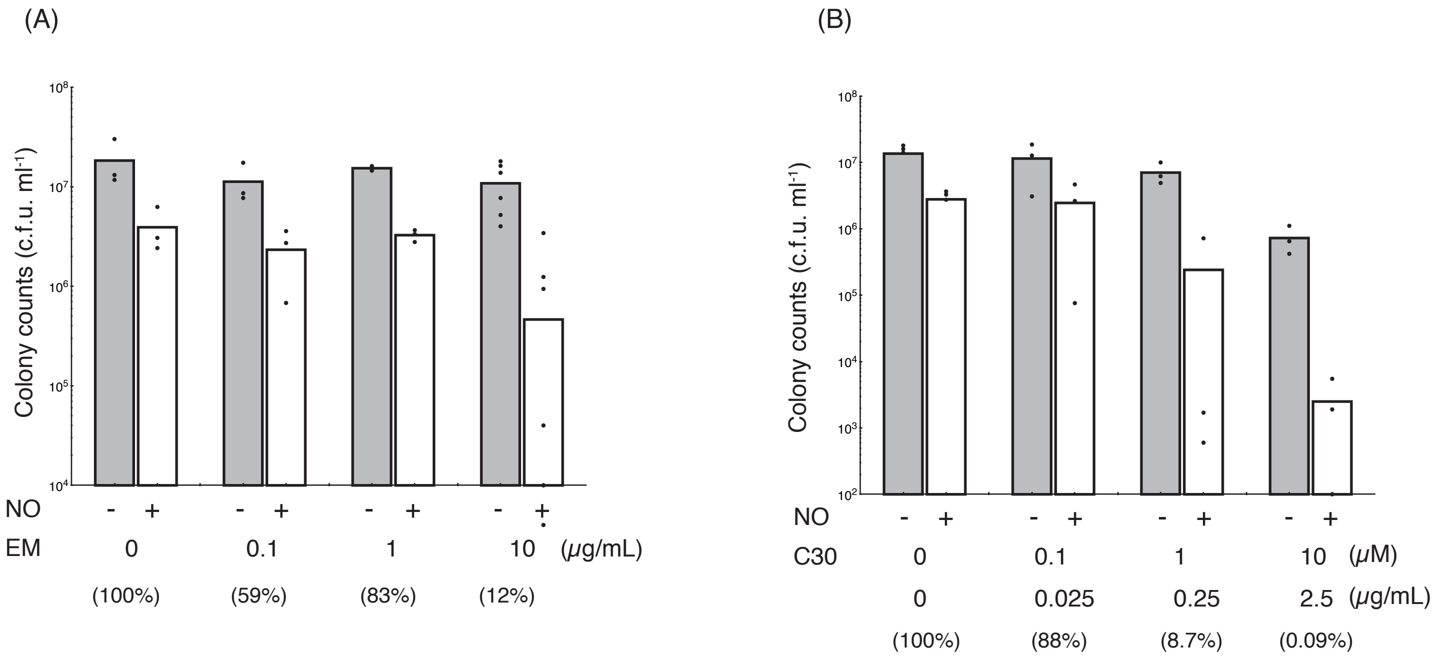

Fig. S2

Effect of EM and C-30 on the sensitivity to nitrosative stress of *P. aeruginosa* PAO1. Bacteria was grown in LB broth containing various concentration of EM or C-30 at 37°C for 18 h. Bacteria were rinsed with saline solution containing 0.1% glucose, 10 mM MOPS (pH 7.0), and the same concentration of EM or C-30. Bacterial suspensions were diluted 1:100 with saline solution containing 0.1% glucose, 10 mM MOPS (pH 7.0), and the same concentration of EM or C-30 with or without 100 μM DETA/NO, and then were incubated at 37°C for 22 h. The number of viable bacteria was determined using bacteria plate counts. Percentages in parentheses represent the ratio to the colony counts treated with the NO donor without EM or C-30. All assays were performed at least three times. Results are expressed as the means and individual data points.

(A): EM; (B): C-30
